# Advancing clinical trial equity through integration of telehealth and decentralized treatment

**DOI:** 10.1093/jncics/pkae050

**Published:** 2024-06-20

**Authors:** Eleanor Brown, George Albert Fisher, Andrew Shelton, Daniel T Chang, Erqi Pollom

**Affiliations:** Department of Radiation Oncology, Stanford University School of Medicine, Palo Alto, CA, USA; Department of Medicine, Stanford University School of Medicine, Palo Alto, CA, USA; Department of Surgery, Stanford University School of Medicine, Palo Alto, CA, USA; Department of Radiation Oncology, Michigan Medicine, Ann Arbor, MI, USA; Department of Radiation Oncology, Stanford University School of Medicine, Palo Alto, CA, USA

## Abstract

Innovative strategies to increase clinical trial accessibility and equity are needed. We conducted a retrospective review of a phase II investigator-initiated trial to determine whether the modification of clinical trial design to decentralize study treatment can improve trial accessibility among underrepresented groups. Sociodemographic characteristics, including area deprivation indices, as well as study site travel distance, time, and costs were compared between enrolled participants who received chemotherapy locally and participants who did not. Participants who received chemotherapy locally lived substantially farther from the study site (median = 95.90 vs 25.20 miles, *P* = .004), faced a greater time burden traveling to the study site (median = 115.00 vs 34.00 minutes, *P* = .002), and had higher travel-related costs for a single trip to the study site (median = $62.81 vs $16.51, *P* = .004). This study highlights opportunities for alleviating financial and time burdens associated with clinical trial participation, promoting equity in clinical research.

**Trial Registration:** ClinicalTrials.gov identifier: NCT04380337.

As it did with many hospitals and research institutions around the world, the COVID-19 pandemic required our National Cancer Institute–designated Comprehensive Cancer Center to adapt and modify existing regulatory policies to allow for the continued participation of patients with cancer in our clinical trials while maintaining patient safety and the scientific integrity of our research protocols. Although these adaptations were primarily driven by the pandemic, some changes had the additional benefit of alleviating the financial and time burdens associated with clinical trial participation.

Financial and time burdens can easily be compounded for patients when they are undergoing cancer treatment (1). Cancer treatment through a clinical trial often incurs additional costs, which include costs associated with transportation, accommodation, time off work, and childcare (2). For example, in the United States, 40% of patients with cancer drive, on average, 60 minutes 1 way to a facility to participate in a clinical trial (3).

We report here an investigator-initiated trial that, in response to the pandemic, restructured its design. This restructuring involved decentralizing a component of the trial, allowing participants to receive their chemotherapy on study with their local oncologist, and integrating telehealth for adverse event reporting. We explore the trial design’s impact on the demographic characteristics of enrolled participants and as a potential mechanism to reducing financial and time burdens associated with trial participation. Written informed consent was obtained from each participant before study enrollment. The trial was approved by the Stanford University Institutional Review Board.

We conducted an investigator-initiated phase II study for patients with rectal cancer that evaluated a regimen of short-course radiation followed by 8 cycles of chemotherapy, with a primary endpoint of clinical complete response rate (ClinicalTrials.gov identifier NCT04380337; Supplementary Methods, available online) (4). Radiation was administered at our institution, and participants could receive chemotherapy closer to home with their local oncologist. This design was permitted because the chemotherapy was a standard-of-care treatment for rectal cancer and not considered a research activity per the Regulations for the Protection of Human Subjects (45 CFR §46), also known as the Common Rule (5). For participants who received chemotherapy locally, the research team held video visits with them before each chemotherapy infusion to review adverse events.

We compared participants’ self-reported demographic data in this trial by location of chemotherapy delivery (local treatment vs nonlocal treatment) using the Fisher exact test and χ^2^ test. The California state decile ranks for Area Deprivation Index (ADI) was calculated from each enrolled participant’s primary residence address, with 1 representing the least disadvantaged areas and 10 representing the most disadvantaged areas (6,7). The ADI of all participants was compared by location of chemotherapy delivery and ethnicity using the Mann-Whitney (Wilcoxon) test. Travel distance and travel time to our institution were calculated from participants’ primary residence address using Google Maps. Travel-related costs were determined by the 2023 Internal Revenue Service mileage reimbursement rate of 65.5 cents per mile traveled, accounting for gas, insurance, and vehicle depreciation (8).

Between May 2020 and April 2023, 37 participants enrolled in the trial. By November 2023, all 37 participants had completed study treatment. Of the 37 enrolled participants, 26 were White (70%), 10 were Asian (27%), 1 was Native Hawaiian/Pacific Islander (3%), none were Black or African American (0%), and 4 were Hispanic/Latino (11%). Demographic and clinical characteristics were similar among participants who received treatment locally and participants who received treatment nonlocally. Demographic and clinical information is detailed in supplementary materials (Supplementary Table 1, available online).

Seventeen participants (46%) received chemotherapy locally. Figure 1 depicts the geographic distribution of the counties in which participants underwent local treatment in relation to our institution. Individuals who received chemotherapy locally lived statistically significantly farther from the study site than individuals who received chemotherapy at the study site (median = 95.90 vs 25.20 miles, *P* = .004). Consequently, participants who received chemotherapy locally faced a statistically significantly greater time burden traveling to our institution than participants who did not (median = 115.00 vs 34.00 minutes, *P* = .002). Similarly, the associated travel-related costs for a single trip to our institution from participants’ primary residence were statistically significantly greater for participants who received chemotherapy locally than participants who did not (median = $62.81 vs $16.51, *P* = .004). Throughout chemotherapy during the study, participants made a minimum of 16 trips to an infusion center. Thus, this cost can be multiplied by 32 to reflect minimum costs associated with round-trip travel during chemotherapy treatment on trial. Furthermore, participants who received chemotherapy locally were more likely to reside in more disadvantaged areas (ADI median = 7.00 vs 2.00, *P* = .038) (Table 1). This trend was also identified when comparing ADI data by ethnicity, with Hispanic/Latino participants being statistically significantly more likely to reside in more disadvantaged areas than non-Hispanic/non-Latino participants (ADI median = 9.00 vs 2.00, *P* = .060). Hispanic/Latino participants traveled statistically significantly farther from their home to our institution than non-Hispanic/non-Latino participants did (median = 127.50 miles vs 33.60 miles, *P* = .022). When comparing the incidence of grade 3 or higher nonhematological toxicity among participants, there was no statistically significant difference between individuals who received treatment locally and individuals who did not (*P* = .860).

**Figure 1.**
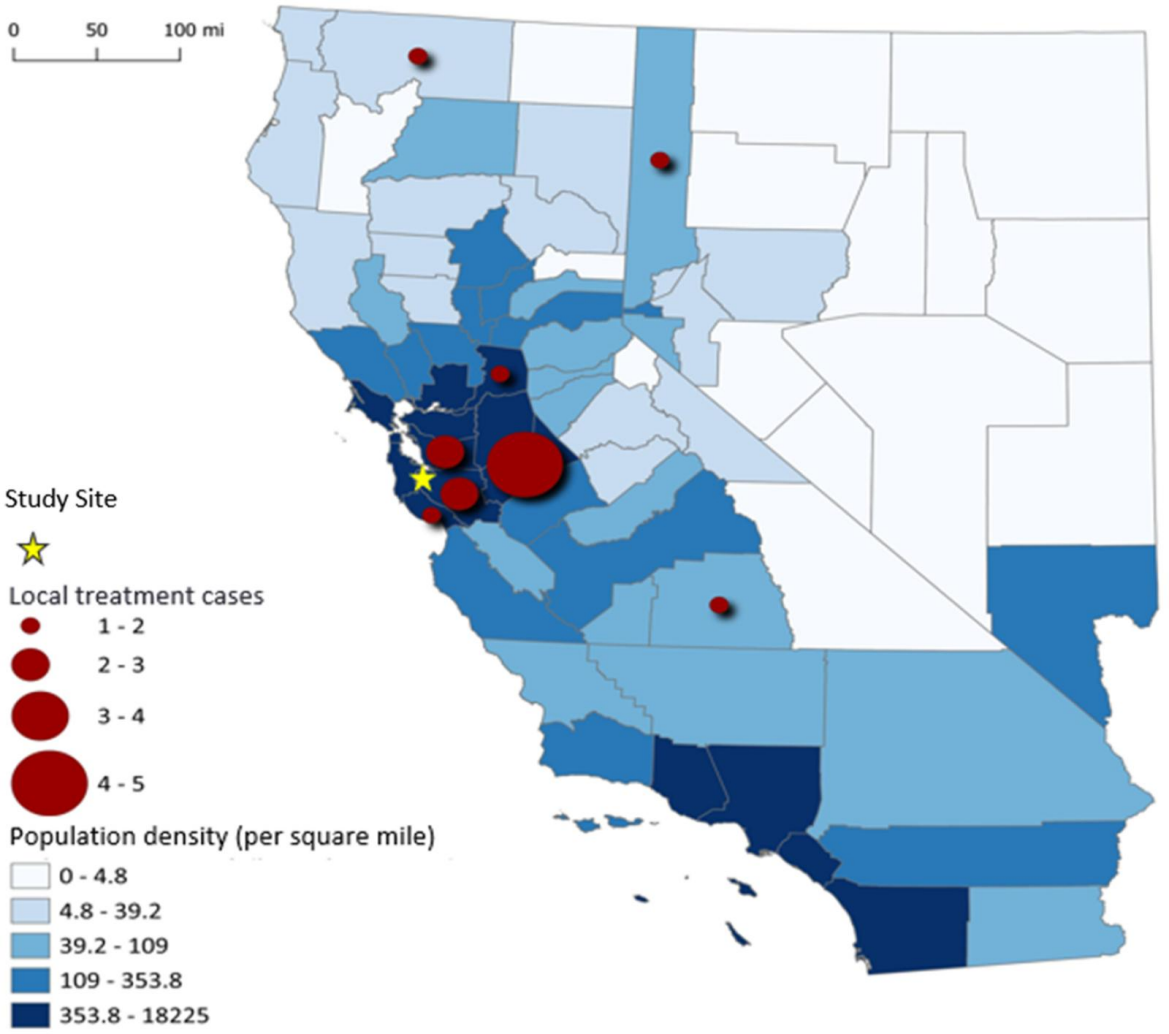
Geographic spread of local oncology clinics in which participants received chemotherapy on study (n = 17).

**Table 1.** Comparison of travel distance, time, and cost, 1 way, to the study site and Area Deprivation Index (ADI) on the basis of location of chemotherapy delivery^a^

	Received chemotherapy locally (n = 17)	Received chemotherapy at study site (n = 20)	*P*
Distance, median (IQR), mi	95.90 (35.10-134.00)	25.20 (15.27-42.85)	.004
Time, median (IQR), min	115.00 (56.00-142.00)	34.00 (27.00-55.25)	.002
Cost, median (IQR), US$	62.81 (22.99-87.77)	16.51 (10.01-28.07)	.004
ADI, median (IQR)	7.00 (2.00-9.00)	2.00 (1.75-3.00)	.038

a IQR = interquartile range

This trial opened to accrual several months into the COVID-19 pandemic, a period marked by transformative changes in health-care delivery and the widespread introduction of telehealth across clinical practices. Adapting to this crisis, we designed a protocol to allow participants to receive their standard-of-care chemotherapy with their local oncologist, with telehealth employed for adverse event reporting. We found that these modifications alleviated many of the financial and time burdens of clinical trial participation for patients who live farther away from our cancer center, particularly individuals who are underrepresented in clinical research.

Although there was no statistically significant difference in the demographic distribution of participants enrolled in this study compared with that of other trials at our institution, we found that individuals who benefited from decentralized chemotherapy resided farther away from our institution and in more disadvantaged areas. Notably, every Hispanic/Latino participant enrolled in this study opted for the convenience of local chemotherapy administration, which allowed for tangible time and cost savings. For individuals who received chemotherapy locally, they would have spent, on average, $2000 on travel alone if they had to travel to our institution for their treatment. To contextualize this added financial burden, this cost is 25.9% of the median monthly income of Stanislaus County, a region where 29.4% of participants who opted for local treatment resided (9).

Telehealth played a pivotal role in facilitating decentralized treatment, enabling study investigators to engage remotely with participants who received chemotherapy locally for assessment of adverse events. It is important to note, however, that the success of this approach assumes a population with the capability to access and participate in telehealth services. At least 1 in every 4 Americans may lack the digital literacy skills or access to devices necessary to participate in video visits (10).

Also integral to the implementation of decentralized treatment was the close collaboration with our governing regulatory bodies to ensure that we were working in accordance with institutional regulations and adhering to the Common Rule (11). To decentralize a research procedure, the institutional regulatory body must first determine whether the institution in which the activity is being conducted is considered “engaged” or “nonengaged” in the research study (5). If an institution is engaged, oversight by an institutional review board may be required. Guidance provided by the US Department of Health and Human Services for making this distinction is outlined in Section III of the Engagement of Institutions in Human Subjects Research (2008) (5).

The chemotherapy in our protocol was considered a nonengaged research activity, given its routine inclusion in community hospitals’ formulary and frequent administration to nonresearch patients. Furthermore, the treating oncologist retained the authority to modify the regimen at their discretion. Importantly, the chemotherapy administration strictly adhered to standard practice, and the community hospital staff were not integrated into the research team. All research-related activities were completed by study investigators at the engaged research institution. A limitation of this study was not collecting data on patient-perceived quality of chemotherapy-associated care. Future studies assessing the impact of decentralized treatment should examine patient-reported outcomes as they relate to treatment satisfaction and financial and time expenditures.

The US Food and Drug Administration is currently taking steps to advance clinical trial decentralization, releasing draft guidelines for decentralizing trials for sponsors, investigators, and other stakeholders (12). Such initiatives provide potential pathways to alleviate the financial and time burdens that patients participating in clinical trials face, which can help to enhance the accessibility and equity of clinical trials.

## Supplementary Material

pkae050_Supplementary_Data

## Data Availability

The data that support the findings of this study are available from the corresponding author upon reasonable request.
