# Supplementary material for: Advancing clinical trial equity through integration of telehealth and decentralized treatment
Source: JNCI Cancer Spectr. 2024 Jun 20;8(4):pkae050. doi: 10.1093/jncics/pkae050 (PMC11240839; doi:10.1093/jncics/pkae050)
Supplement: pkae050_Supplementary_Data [file pkae050_supplementary_data.zip › Supplementary Table 1_2.docx]

**Supplementary Table 1:** Demographic and clinical characteristics of local treatment cases vs. non-local treatment cases (N=37)

|  | **Total participants on trial (N=37)** | **Local treatment (n=17)** | **Non-local treatment (n=20)** | ***P* value^b^** |
| --- | --- | --- | --- | --- |
| **Sex, n (%)** | |  |  | 0.75 |
| Female | 13 (35.1%) | 5 (29.4%) | 8 (40.0%) |  |
| Male | 24 (64.9%) | 12 (70.6%) | 12 (60.0%) |  |
| **Age (years), n (%)** | |  |  | 0.97 |
| 20-39 | 6 (16.2%) | 3 (17.6%) | 3 (10.0%) |  |
| 40-69 | 24 (64.9%) | 11 (64.7%) | 13 (70.0%) |  |
| 70+ | 7 (18.9%) | 3 (17.6%) | 4 (20.0%) |  |
| **Race, n (%)** | |  |  | NA |
| Asian | 10 (27.0%) | 2 (11.8%) | 8 (40.0%) |  |
| Black or African American | 0 (0.0%) | 0 (0.0%) | 0 (0.0%) |  |
| Native Hawaiian or Other Pacific Islander | 1 (2.7%) | 0 (0.0%) | 1 (5.0%) |  |
| White | 26 (70.3%) | 15 (88.2%) | 11 (55.0%) |  |
| Other^a^ | 0 (0.0%) | 0 (0.0%) | 0 (0.0%) |  |
| **Ethnicity, n (%)** | |  |  | 0.07 |
| Hispanic or Latino | 4 (10.8%) | 4 (23.5%) | 0 (0.0%) |  |
| Non-Hispanic | 33 (89.2%) | 13 (76.5%) | 20 (100.0%) |  |
| **ECOG** |  |  |  | 0.75 |
| 0 | 26 (70.3%) | 11 (64.7%) | 15 (75.0%) |  |
| 1 | 11 (29.7%) | 6 (35.3%) | 5 (25.0%) |  |
| **Clinical T Stage** |  |  |  | 0.18 |
| cT2 | 3 (8.1%) | 0 (0.0%) | 3 (15.0%) |  |
| cT3 | 26 (70.3%) | 14 (82.4%) | 12 (60.0%) |  |
| cT4 | 8 (21.6%) | 3 (17.6%) | 5 (25.0%) |  |
| **Clinical N Stage** |  |  |  | 0.25 |
| cN0 | 7 (18.9%) | 5 (29.4%) | 2 (10.0%) |  |
| cN1 | 15 (40.5%) | 5 (29.4%) | 10 (50.0%) |  |
| cN2 | 15 (40.5%) | 7 (41.2%) | 8 (40.0%) |  |
| ^a^ includes participants identifying as American Indian or Alaska Native or More than One Race  ^b^ Fisher's exact test was used to calculate Age, Race, Ethnicity, Clinical T and N Stage; chi-square test was used to calculate Sex and ECOG | | | | |
