# Supplementary material for: Advancing clinical trial equity through integration of telehealth and decentralized treatment
Source: JNCI Cancer Spectr. 2024 Jun 20;8(4):pkae050. doi: 10.1093/jncics/pkae050 (PMC11240839; doi:10.1093/jncics/pkae050)
Supplement: pkae050_Supplementary_Data [file pkae050_supplementary_data.zip › Supplementary Material Research Protocol.docx]

**Protocol IRB-56027 OnCore#COR0019 NCT- 04380337**

**Phase II Trial of Organ Preservation Program Using Short-Course Radiation and FOLFOXIRI for Rectal Cancer (SHORT-FOX)**

**Protocol Director / Principal Investigator**

Erqi Pollom, MD, MS

Associate Professor, Radiation Oncology
875 Blake Wilbur
Stanford, CA 94305

Coordinating Center

Stanford Cancer Institute

875 Blake Wilbur Drive

Stanford, CA 94305

**Co-Principal Investigators**

George Fisher, MD, PhD

Professor, Medical Oncology

875 Blake Wilbur

Stanford, CA 94305

Andrew Shelton, MD

Clinical Professor, General Surgery

300 Pasteur Dr

Stanford, CA 94305

**SRC Initial Approval Date: 30 March 2020**

Protocol Version: 15

Protocol Version Date: 3 February 2023

**TABLE OF CONTENTS**

PROTOCOL SYNOPSIS 7

SCHEMA 8

LIST OF ABBREVIATIONS AND DEFINITION OF TERMS 9

1. OBJECTIVES 10

1.1. Primary Objective 10

1.2. Secondary Objectives 10

2. BACKGROUND 10

2.1 Study Disease 10

2.2 Study Agent/Device/Procedure 11

2.3 Rationale 15

2.4 Study Design 16

2.5 Correlative Studies Background 16

3. PARTICIPANT SELECTION AND ENROLLMENT PROCEDURES 16

3.1 Participant Eligibility Checklist 16

3.2 Informed Consent Process 20

3.3 Randomization Procedures 20

3.4 Study Timeline 20

4. TREATMENT PLAN 20

4.1 Pretreatment Studies 20

4.2 Treatment Schedule 20

4.3 General Concomitant Medication and Supportive Care Guidelines 24

4.3 Criteria For Removal From Studies 24

4.5 Alternatives 25

5. INVESTIGATIONAL AGENT/DEVICE/PROCEDURE INFORMATION 25

6. DOSE MODIFICATIONS 25

6.1 Chemotherapy 25

6.2 Radiation Therapy 29

7. ADVERSE EVENTS AND REPORTING PROCEDURES 30

7.1 Potential Adverse Events 30

7.2 Adverse Event Reporting 34

8. CORRELATIVE/SPECIAL STUDIES 39

8.1 ctDNA Study 39

9. STUDY CALENDAR 40

10. MEASUREMENT 41

10.1 Primary Outcome measures 41

10.2 Secondary Outcome Measures 41

10.3 Exploratory Outcome Measures 42

11. REGULATORY CONSIDERATIONS 43

11.1 Institutional Review of Protocol 43

11.2 Data and Safety Monitoring Plan 43

11.3 Data Management Plan 43

12. STATISTICAL CONSIDERATIONS 44

12.1 Statistical Design 44

12.2 Interim analyses 44

12.3 Descriptive Statistics and Exploratory Data Analysis 44

12.4 Primary Analysis 44

12.5 Secondary Analysis 44

12.6 Sample Size 45

12.7 Criteria for future studies 46

13. REFERENCES 47

APPENDICES 54

APPENDIX A: Definition of Menopausal Status 55

hrqol Documentation 56

APPENDIX B: Mskcc Bowel Instrument 56

APPENDIX C: Low Anterior Resection Syndrome 60

APPENDIX D: Stoma QOL 62

APPENDIX E: IIFE-5 Instrument 64

APPENDIX F: FSFI-6 Instrument 65

APPENDIX G: International Prostate Symptom Score 66

APPENDIX H: EQ-5D-5L Instrument 68

# PROTOCOL SYNOPSIS

| TITLE | Phase II Trial of Organ Preservation Program Using Short-Course Radiation and FOLFOXIRI for Rectal Cancer (SHORT-FOX) |
| --- | --- |
| STUDY PHASE | Phase II |
| INDICATION | Treatment |
| INVESTIGATIONAL PRODUCT OR PROCEDURE | Organ preservation approach using short course radiation followed by FOLFOXIRI for patients with non-metastatic, >T2N0 or low T2N0 rectal cancer |
| PRIMARY OBJECTIVE | To assess clinical complete response of an organ preservation approach using short course radiation followed by intensified chemotherapy |
| SECONDARY OBJECTIVES | To assess safety in all enrolled patients, local regrowth rate and other cancer specific outcomes (disease-free survival, colostomy-free survival and overall survival), longitudinal health-related quality of life of this organ preservation approach |
| TREATMENT SUMMARY | Patients will undergo short-course radiation followed by 4 months of FOLFOXIRI. Those who achieve a clinical complete response will be considered for organ preservation approach. All other patients will receive standard of care total mesorectal excision (TME). |
| SAMPLE SIZE | 37 patients |
| STATISTICAL CONSIDERATIONS | Sample Size Calculation: We plan to use an optimal Simon 2 stage design and enroll 37 patients with an interim analysis at 17 patients for futility.  Primary Endpoint: Clinical complete response assessed 8 (+/-4) weeks after completion of chemotherapy.  Secondary Endpoints: Toxicity, local regrowth rate, disease-free survival, colostomy-free survival, and overall survival assessed until study completion.  Exploratory Endpoint: Health-related quality of life assessed until study completion. |

# SCHEMA

#
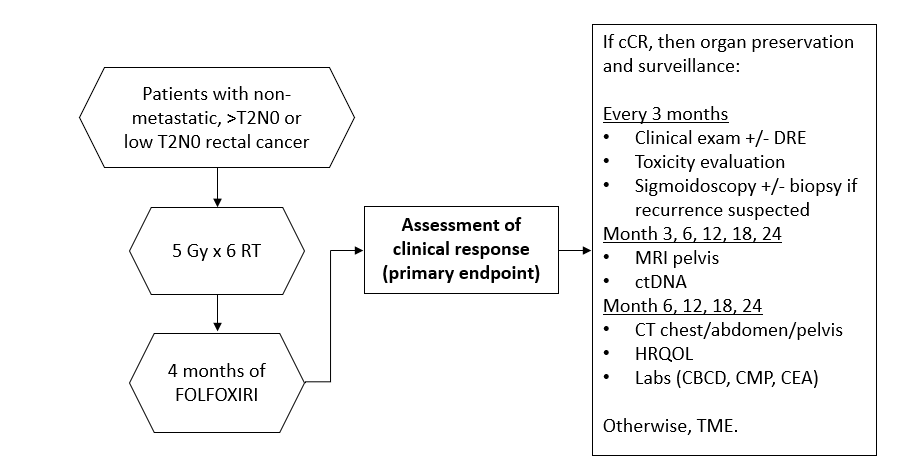
LIST OF ABBREVIATIONS AND DEFINITION OF TERMS

| 5FU | 5-fluorouracil |
| --- | --- |
| AE | Adverse effect |
| APR | abdominoperineal resection |
| CAPOX | capecitabine and oxaliplatin |
| cCR | Clinical complete response |
| CRT | Chemoradiotherapy |
| ctDNA | Circulating tumor DNA |
| DFS | Disease-free survival |
| DRE | Digital Rectal Exam |
| FOLFOX | leucovorin, 5-fluorouracil, and oxaliplatin |
| FOLFOXIRI /mFOLFIRINOX | leucovorin, 5-fluorouracil, oxaliplatin, and irinotecan |
| HRQOL | Health-related quality of life |
| mrTRG | Magnetic Resonance Tumor Regression Grade |
| OS | Overall survival |
| pCR | Pathologic complete response |
| RT | Radiation |
| SCRT | Short-course radiation |
| TAE | Transanal full-thickness excision |
| TME | Total mesorectal excision |
| TNT | Total neoadjuvant therapy |
| WW | Watch and wait |
| XELOX | oxaliplatin, capecitabine |

# 1. OBJECTIVES

## 1.1. Primary Objective

- To assess clinical complete response (cCR) of an organ preservation approach for non-metastatic, >T2N0 or low T2N0 rectal cancer using short course radiation (SCRT) followed by intensified chemotherapy.

## 1.2. Secondary Objectives

- To assess safety in all patients and local regrowth rate and other cancer specific outcomes (disease-free survival [DFS], colostomy-free survival and overall survival [OS]) among patients assigned to organ preservation approach.
- To assess longitudinal functional outcomes and health-related quality of life [HRQOL] of this organ preservation approach.

# 2. BACKGROUND

## 2.1 Study Disease

Colorectal cancer represents the 3^rd^ most common cancer and the 3^rd^ leading cause of cancer death in both men and women the United States. In 2019, there was an estimated incidence of 145,600 new cases, 44,200 of which were rectal cancer^1^.  Locally advanced rectal cancer has historically been associated with a high rate of recurrence in the pelvis following surgery^2^, which can result in significant morbidity and reduce survival^3^.

The German Rectal Study set the standard of care as preoperative chemoradiotherapy (CRT) followed by total mesorectal excision (TME) where 823 patients were randomized to preoperative or postoperative CRT with continuous infusion 5-fluorouracil (5FU)^4^. In the preoperative treatment arm, radiation (RT) was to 50.4 Gy at 1.8 Gy/fraction to the pelvis. In the postoperative treatment arm, RT was to 50.4 Gy to the pelvis followed by a 5.4 Gy boost to the primary tumor. The study found a statistically significant decrease in incidence of local relapse with preoperative versus postoperative CRT (6% vs. 13%, p=0.006) and no difference in 5-year OS (76% vs. 74%, p=0.8). The pathologic complete response (pCR) rate in the preoperative CRT group was 8%. Patients initially thought to require an abdominoperineal resection (APR) who had preoperative CRT had a higher rate of sphincter-preserving surgery than those who had postoperative CRT (39% vs. 19%, p=0.004), indicating the success of tumor downstaging. Additionally, patients who received preoperative CRT had less Grade 3 or 4 toxicity than those who received postoperative CRT, both with respect to acute toxicity (27% vs. 40%, p=0.001) and long-term toxicity (14% vs. 24%, p=0.01).

Thus, in the United States, locally advanced rectal cancer is standardly treated with preoperative CRT followed by TME^4^. Local recurrence rates with this approach are approximately 5%^5–7^, and are even lower (1%) among patients who achieve a pCR^5,8^. However, TME is associated with significant morbidity, including wound complications, sexual dysfunction, urinary retention, anastomotic leaks and strictures. While abdominoperineal resection has been shown to be associated with worse body image, micturition symptoms, and sexual function compared to sphincter-sparing surgery, 50-90% of patients who undergoing sphincter-sparing surgery will have bowel dysfunction, including low anterior resection syndrome which is characterized by clustering stools, soiling and fecal incontinence^9^.

## 2.2 Study Agent/Device/Procedure

**2.2.1 Organ preservation**

Given the excellent locoregional control achieved with aggressive trimodality treatment, there is interest in de-intensifying local therapies to reduce treatment-associated morbidity and long-term sequela. Early data suggest that patients who achieve a cCR after CRT may represent a favorable cohort who may be safely observed^10–18^ or locally excised^19–22^.

The first publication by Habr-Gama et al. compared a group of patients managed with watch and wait (WW) following a cCR after CRT with a group of patients who underwent neoadjuvant CRT with a pCR after surgery^18^. In the WW group, 5-year OS and DFS were 100% and 92%, respectively, with only 2 of 71 patients (2.8%) having local regrowth. In the resection group, both 5-year OS and DFS were lower, at 88% and 83%, respectively. Since then, numerous publications have demonstrated high rates of sustained local control and excellent OS with a WW approach. Recently, an international WW database has confirmed that in a large international dataset of 800 patients managed with WW at 47 centers in 15 countries, the long-term outcomes remain favorable with a 5-year DFS and OS of 94% and 85%, respectively^10^. A prospective randomized trial is currently underway by the Sao Paolo group randomizing clinical complete responders to TME versus WW, although using a different regimen than what this study proposes.

More conservative surgery has also been explored. In GRECCAR 2, patients with rectal tumors 4 cm or smaller at 8 cm from the anal verge were treated with preoperative therapy^21^. Those who had a good clinical response (scar <2 cm) were randomized to TME or local excision. 148 patients (80%) were randomized. 60% of the patients randomized to local excision were found to be ypT0-1 and were followed while the rest underwent completion TME (for ypT2/3 disease or R1 resection). Local recurrence was only 5%, and there were no differences in local recurrence rates, DFS or OS between the two groups. Local excision has shown to be a reasonable treatment alternative for small tumors only, as patients on GRECCAR 2 who had ypT2+ disease had high rates of nodal positivity (8-40%) which would not be addressed with local excision alone. Further, a Polish trial showed high rates of local recurrence in those patients with positive margins or ypT2-3 disease after CRT treated with local excision alone^23^.

These approaches may thereby improve short- and long-term quality of life, as TME is associated with perioperative morbidity and mortality, sexual, urinary, and bowel dysfunction, low anterior resection syndrome, and/or permanent ostomy^24–26^. However, there are important challenges with these approaches. First, there is no consensus on the definition of cCR. Memorial Sloan Kettering Cancer Center has proposed criteria for evaluating tumor response using a 3-tiered assessment of response/regression (cCR, near cCR, incomplete response) based on results of endoscope, digital rectal exam, and MRI^27^. This has not yet been validated. Further, a uniform surveillance protocol following organ preservation is also critical. The promising early data of WW and local excision represent highly selected patients who undergo careful surveillance, so these strategies do require a commitment to actively follow these patients carefully. Although a uniform protocol has not yet been established, these patients have typically been followed with digital rectal exam, flexible endoscopy, and MRI for a total of 5 years, with more frequent surveillance in the first 2 years. Potential tumor-specific early biomarkers of recurrence such as circulating tumor DNA are being investigated so that they can be incorporated into the surveillance strategy following organ preservation.

**2.2.2 Total neoadjuvant therapy (FOLFOXIRI)**

Despite excellent local control, rates of distant metastasis in this population can exceed 25%.^5,28,29^ To address micrometastases and the risk of distant disease, chemotherapy that typically has been delivered following surgery is delivered prior to surgery in an increasingly used approach called “total neoadjuvant therapy (TNT)”^30,31^. Delivering all of the chemotherapy before rather than after surgery may also reduce interval to diverting loop ileostomy takedown and increase treatment compliance^32^. Finally, as only up to 15-20% of patients have an adequate response following standard CRT, TNT can increase tumor response and likelihood of patient eligibility for non-operative management.

Findings from several phase 2 trials have shown enhanced tumor response with TNT. On a phase 2 trial^30^, patients with poor-risk rectal cancer (tumor extending to within 1 mm of or beyond the mesorectal fascia, T3 low lying tumor at or below the levators, T4 tumor, N2 disease, or tumor extending 5 mm or more into the perirectal fat) received four 21-day cycles of oxaliplatin and capecitabine (CAPOX), for a total of 12 weeks of chemotherapy, followed by 54 Gy of pelvic CRT with concurrent capecitabine. 20% of patients achieved a pCR with this regimen. During neoadjuvant chemotherapy, grade 3-5 toxicities included anemia (1%), neutropenia (1%), thrombocytopenia (1%), diarrhea (10%), cardiac or thromboembolic events (9%), lethargy (7%), nausea and vomiting (3%), hand-foot syndrome (3%) and infection (1%). There was no stomatitis, febrile neutropenia or neuropathy. During CRT, grade 3-5 toxicities involved skin (42%), lower gastrointestinal (3%) and genitourinary (3%). On another phase II trial, patients with high-risk rectal cancer were randomized to four weeks of CAPOX followed by CRT or the same regimen plus weekly cetuximab^33^. cCR and pCR were 18% and 18%, respectively, for the CAPOX arm, and not improved with the addition of cetuximab. Grade 3-5 toxicities with CAPOX included febrile neutropenia (1%), diarrhea (9%), lethargy (10%), nausea and vomiting (2%), hand-foot syndrome (1%); grade 3-5 toxicities during CRT following CAPOX included diarrhea (1%) and hand-food syndrome (1%).

A recent systematic review and meta-analysis examined 28 studies looking at outcomes of TNT for locally advanced rectal cancer; these included a total of 2,688 patients treated with TNT. The pooled pCR rate was 22.4%. Ten of the studies compared pCR rates for treatment with TNT versus standard CRT; pooled analysis showed that TNT increased the odds of pCR by 39% (OR 1.40, 95% CI 1.08-1.81, p=0.01). Seven studies compared survival outcomes; patients treated with TNT had significantly better OS (HR 0.73, 95% CI 0.59-0.9, p=0.004)^34^.

In metastatic colorectal cancer, a triplet regimen with 5FU, oxaliplatin and irinotecan (FOLFOXIRI) has been shown to be superior to standard doublet chemotherapy^35^. This intensified regimen has been explored in combination with bevacizumab and CRT in rectal cancer in order to maximize response^36^. This study enrolled 49 patients with T4 or high-risk T3 rectal cancer; they received 6 cycles of FOLFOXIRI plus bevacizumab followed by CRT and then surgery 8 weeks later. pCR with this regimen was 36%. Grade 3-5 toxicities during induction chemotherapy and CRT included thrombocytopenia (2%), neutropenia (42%), febrile neutropenia (4%), diarrhea (12%), stomatitis (4%), asthenia (6%), hypertension (2%), aminotransferases elevation (2%) and rectovaginal fistula (2%). One patient died due to bowel perforation (a known bevacizumab-related adverse event) and sepsis after the first cycle of chemotherapy and another patient discontinued induction treatment after acute kidney injury (followed by complete recovery). One patient had pneumonia after chemotherapy and went directly to surgery.

## 2.2.3 Short course radiation (SCRT)

While the pivotal German Rectal trial^4^ established preoperative CRT as standard of care for locally advanced rectal cancer in the United States, multiple trials have demonstrated the efficacy of SCRT^5,24,37–40^. The advantages of SCRT over long-course CRT include excellent compliance, cost-effectiveness, and better integration with systemic therapy. While there were initial concerns for lack of downstaging of bulky tumors and inferior outcomes with low-lying tumors with SCRT^40,41^, the addition of a delay (during which chemotherapy can be delivered) prior to TME has resulted in comparable outcomes with SCRT as CRT^39,42^.

The Polish Colorectal Study group trial randomized patients with cT3 or cT4 rectal cancer (>60% of patients had T4 tumors; over half of patients had tumors <5 cm from anal verge) to either preoperative SCRT (5 x 5 Gy) followed by 3 cycles of FOLFOX-4 or CRT (50.4 Gy of RT in 28 fractions of 1.8 Gy each concomitantly with oxaliplatin, boluses of fluorouracil, and folinic acid).^39,43^ These regimens were chosen to ensure equivalent treatment time and interval from initiation of RT to TME. The proportion of patients with a pCR was greater among patients who received SCRT and consolidation chemotherapy than among patients who received CRT, but the difference did not reach statistical significance (16% versus 12%, p=0.17). There was no difference in DFS, OS or long-term complications.

Washington University School of Medicine (St. Louis, MO) reported good overall response to a preoperative regimen that comprised SCRT followed by 4 cycles of FOLFOX with TME performed 4 to 9 weeks after preoperative chemotherapy. At surgery, 70% of patients had ypT0-2 residual disease, including 25% with pCR^44^. A matched pair analysis from the same group showed that compared to standard preoperative CRT, SCRT with TNT (modified FOLFOX x 6 cycles) had higher T- downstaging and superior distant metastasis-free survival and DFS. Grade 3-5 hematologic toxicities were higher with SCRT and TNT (22% versus 0%, p<0.001). Grade 3-5 gastrointestinal toxicities were lower with SCRT and TNT compared to CRT, although not statistically significant (6% v 13%, p=0.24)^45^.

The RAPIDO trial compared standard pre-operative CRT with pre-operative SCRT followed by 6 cycles of CAPOX (capecitabine and oxaliplatin) for patients with locally advanced rectal cancers, with the hypothesis that SCRT with neoadjuvant chemotherapy increases DFS and OS without compromising local control^46^.

Preliminary results of this trial showed that compliance was good in both treatment arms^47^.

**2.2.4** **Radiation dose-escalation**

Escalating the dose of RT to gross tumor prior to surgical resection can also help to improve rates of cCR. In a prospective observational study, patients with cT2-3 cN0-1 rectal tumors within 6 cm of the anal verge were treated with CRT using concurrent tegafur-uracil chemotherapy and higher RT doses (60 Gy to primary tumor using external beam RT followed by a 5 Gy endorectal brachytherapy boost)^17^. With this RT dose-escalated regimen, cCR was 78%.

**2.2.5 Timing of surgery following radiation**

Extending the time to surgery following RT can help to further improve rates of cCR and pCR. The Lyon R90-01 trial showed improved clinical response (72 versus 52%) and pCR/near pCR (26 versus 10%) with a 6-8 week delay to TME compared to a 2 week delay^48^ . There was no difference in morbidity, local control or OS. The Stockholm III trial also showed that delay to TME following SCRT (4-8 weeks versus 1 week) was associated with improved pCR and reduced post-operative complications^42^.

There is a concern of increased complications with a prolonged delay. The GRECCAR-6 trial shows that waiting >11 weeks (versus 7 weeks) after CRT for TME did not increase rate of pCR and was associated with increased surgical morbidity (44.5 versus 32%) as a result of increased medical complications (33 versus 19%) and worse quality TME (complete mesorectum 79 versus 90%)^49^ . Thus, TME has standardly been performed at around 6-10 weeks after CRT.

However, The Timing of Rectal Cancer Response to Chemoradiation Trial, which examined outcomes with delivering 2, 4, or 6 cycles of FOLFOX after preoperative CRT (thereby extending the delay to TME after RT to as long as 19 weeks), did not show increased adverse events or surgical complications with longer delay to TME. In fact, this study showed increased pCR rates with increasing FOLFOX cycles and subsequently longer delay to TME, with the group receiving 6 cycles of FOLFOX achieving a 38% pCR rate^20^. Additionally, recent data from a randomized phase II trial showed that upfront CRT followed by 3 cycles of FOLFOX compared to 3 cycles of FOLFOX followed by CRT resulted in better compliance with CRT and did not increase surgical morbidity^50^ .

Thus, the optimal chemotherapy and RT sequencing within TNT is still to be determined. The OPRA trial is a phase II multicenter randomized trial that recently completed accrual and compared two different non-operative management approaches for locally advanced rectal cancer: 16-18 weeks of chemotherapy followed by CRT versus CRT followed by 16-18 weeks of chemotherapy^27^. Those patients who did not achieve a cCR would undergo TME after 18 weeks following RT.

**2.2.6. Determination of clinical complete response**

Currently, response following CRT is currently best assessed by a dedicated multidisciplinary team and includes digital rectal exam (DRE), flexible endoscopy and MRI.^51^  While there are no formal, evidence-based criteria for classifying response following CRT, MSKCC has proposed a three-tiered regression schema that was used the recently completed OPRA Phase II randomized organ preservation trial^27^. A 5-point MRI tumor regression grade (mrTRG) has also been developed and is being used in ongoing trials^52,53^ . It has been found to be reliable and reproducible between multiple independent radiologist and validated against both pathology and survival outcomes^54-56^ . PET/MRI may also be helpful for restaging but further research is still required to provide standardized parameters before PET/MRI can be implemented into clinical practice^57^. Biomarker development is needed to better identify patients who achieve a cCR and may be appropriate candidates for treatment de-escalation in order not to jeopardize the excellent cure rates with standard of care therapy.

With current tools, up to 30% of complete responders are not identified at clinical response assessment^51,58^. Thus, in selected patients, an extending waiting interval could be considered for patients who show a “near cCR” at first response assessment to allow for further regression and help determine whether or not there is a cCR^59^ .

## 2.3 Rationale and Novelty

This study will determine whether intensifying both chemotherapy and radiotherapy can lead to higher rates of cCR, compared to historical controls (~20%), thereby leading to more patients being eligible for organ preservation strategies. Those who achieve a cCR will be managed with organ preservation and surveillance, per multi-disciplinary review, following this regimen.

Our study is novel because no study of watch-and-wait has yet incorporated a regimen that includes short-course radiation. This builds upon/complements studies like 1) RAPIDO, which investigates short-course radiation prior to *surgical resection*, 2) OPRA, which investigates timing of *long-course* radiation with respect to chemotherapy, for watch-and-wait, and 3) GRECCAR 12 (NCT02514278), which investigates the FOLFIRINOX regimen in combination with *long-course* radiation for watch-and-wait.

We believe short-course radiation can potentially improve upon these approaches, as measured by clinical complete response, because it minimizes the time a patient is off multi-agent systemic chemotherapy in order to receive 5-6 weeks of long-course radiation.

## 2.4 Study Design

This is a single‑arm, open-label, non-randomized study and uses a historical control to assess treatment efficacy.

## 2.5 Correlative Studies Background

Circulating tumor DNA (ctDNA) analysis from the peripheral blood can be used as a noninvasive method for tumor interrogation and monitoring. ctDNA is detectable in over 75% of patients with localized colorectal cancers^60^ and has been shown in the post-operative setting to be an indicator of minimal residual disease and predict radiologic recurrence with better sensitivity than CEA measurement.^61,62^ Biomarker development is needed to better select patients for treatment de-escalation as well as monitor patients for recurrence in order not to jeopardize the excellent cure rates following standard of care therapy. We plan to assess the association between ctDNA levels and cCR among all patients and local regrowth and DFS among patients who pursue organ preservation. We hypothesize that ctDNA levels and change of ctDNA levels can help predict clinical outcomes.

# 3. PARTICIPANT SELECTION AND ENROLLMENT PROCEDURES

## **3.1** Participant Eligibility Checklist

| Protocol Title: | **Phase II Trial of Organ Preservation Program Using Short-Course Radiation and FOLFOXIRI for Rectal Cancer (SHORT-FOX)** |
| --- | --- |
| Protocol Number: | **IRB56027/COR0019** |
| Protocol Version Number/Date: |  |
| Principal Investigator: | **Erqi Pollom, MD, MS** |

**Subject Information:**

| Subject Name/MRN: |  |
| --- | --- |
| Gender:  Male  Female | |

|  | **Eligibility Criteria** | **Window/**  **Last Date to Enroll** | **Supporting Documentation** | **Tab Label** | **Met?**  **Y/N or N/A** |
| --- | --- | --- | --- | --- | --- |
| 1 | Pathologically (histologically or cytologically) proven diagnosis of adenocarcinoma of rectum requiring total mesorectal excision as deemed by multidisciplinary evaluation | Within 180 days  Bx: 180 days | Diagnosis:  Bx date: |  |  |
| 2 | At least 18 years of age | N/A | Age: |  |  |
| 3 | For women of childbearing potential or who are not postmenopausal (see Appendix B for Definition of Menopausal Status), a negative urine pregnancy test must be done. Also, women of childbearing potential and men must agree to use adequate contraception (hormonal or barrier method of birth control; abstinence) for the duration of study participation and for up to 4 weeks following the study. Should a woman become pregnant or suspect she is pregnant while participating in this study, she should inform her treating physician immediately. | Within 45 days of enrollment  Pregnancy Test: 45 days | Result of pregnancy test:  Pregnancy Test date: |  |  |
| 4 | ECOG Performance Status of 0-2 | Within 45 days of enrollment  ECOG: 45 days | ECOG:  ECOG date: |  |  |
| 5 | Ability to understand and the willingness to personally sign the written IRB‑approved informed consent document | N/A | ICF date: |  |  |
| 6 | Patients must have acceptable organ and marrow function as defined below:   - ANC >1,500/uL - Hg > 8.0 g/dL; if blood transfusion is performed for achieving adequate hemoglobin level, the level should stay above goal for at least 1 week after transfusion - Platelets >100,000/uL - Total bilirubin <1.5X normal institutional limits - AST(SGOT) / ALT(SGPT) < 3X upper limit of normal - Creatinine <1.5X upper limit of normal or CrCL >50 by Cockcoft-Gault   Patients must have CEA:   - CEA | Within 45 days of enrollment  CBCD: 45 days  CMP: 45 days  CEA: 45 days | CBCD date:  ANC  Hg  PLT  CMP date:  Bilirubin  AST  ALT  Creatinine  CEA date:  CEA |  |  |
| 7 | Clinical stage >T2N0 or low T2N0 rectal cancer (AJCC, 8th ed.) including no metastases based on the following diagnostic workup:  • General history and physical examination with DRE (if deemed appropriate by treating physician) within 45 days prior to enrollment  • Sigmoidoscopy within 90 days prior to enrollment  • The following imaging studies are required within 45 days prior to enrollment:  a) CT chest/abdomen/pelvis  b) MRI Pelvis | H&P: 45 days  Scope: 90 days  CT CAP: 45 days  MRI Pelvis: 45 days | TNM:  H&P: date:  Scope date:  CT CAP date:  MRI Pelvis date: |  |  |
|  | | | | | |
|  | **Ineligibility Criteria** |  |  |  |  |
| 1 | Prior pelvic RT or chemotherapy for rectal cancer | N/A |  |  |  |
| 2 | Upper T2N0 rectal cancers eligible for sphincter-preservation surgery | N/A |  |  |  |
| 3 | Use of other investigational agents | N/A |  |  |  |
| 4 | Ongoing or active infections requiring systemic antibiotic treatment or uncontrolled intercurrent illness including but not limited to symptomatic congestive heart failure, unstable angina pectoris, cardiac arrhythmia, or psychiatric illness/social situations that would limit compliance with study requirements. | N/A |  |  |  |
| 5 | Any concurrent malignancy other than non-melanoma skin cancer or carcinoma in situ of the cervix. Patients with any previous malignancy without evidence of disease for >3 years will be allowed to enter the trial. | N/A |  |  |  |
| 6 | Known hypersensitivity to 5-FU compounds | N/A |  |  |  |
| 7 | Pregnant and breastfeeding women are excluded. Women of child-bearing potential who are unwilling or unable to use an acceptable method of birth control to avoid pregnancy for the entire study period and for up to 4 weeks after the study are excluded. (This applies to women who have experienced menarche and have not undergone successful surgical sterilization or are not postmenopausal). | N/A |  |  |  |
| 8 | Because patients with immune deficiency are at increased risk of lethal infections when treated with marrow-suppressive therapy, known HIV-positive patients with detectable viral loads and/or receiving combination anti-retroviral therapy are excluded from the study | N/A |  |  |  |
| 9 | Primary unresectable rectal cancer (tumor invading adjacent organs and en bloc resection will not achieve negative margins) | N/A |  |  |  |

*All subject files must include supporting documentation to confirm subject eligibility. The method of confirmation can include, but is not limited to, laboratory test results, radiology test results, subject self-report, and medical record review.

**Statement of Eligibility**

By signing this form of this trial I verify that this subject is [ **eligible** /  **ineligible**] for participation in the study. This study is approved by the Stanford Cancer Institute Scientific Review Committee, the Stanford IRB, and has finalized financial and contractual agreements as required by Stanford School of Medicine’s Research Management Group.

| Treating Physician Signature: | Date: |
| --- | --- |
| Printed Name: | |

| Secondary Reviewer Signature: | Date: |
| --- | --- |
| Printed Name: | |

| Study Coordinator Signature: | Date: |
| --- | --- |
| Printed Name: | |

## 3.2 Informed Consent Process

All participants must be provided a consent form describing the study with sufficient information for participants to make an informed decision regarding their participation. Participants must sign the IRB approved informed consent prior to participation in any study specific procedure. The participant must receive a copy of the signed and dated consent document. The original signed copy of the consent document must be retained in the medical record or research file.

## 3.3 Randomization Procedures

Not applicable.

## 3.4 Study Timeline

**Primary Completion:**

The study will reach primary completion 60 months from the time the study opens to accrual.

**Study Completion:**

The study will reach study completion 84 months from the time the study opens to accrual.

# 4. TREATMENT PLAN

- 1. **Pretreatment Studies**

The following will be completed after enrollment and before treatment begins

- HRQOL
- ctDNA
- AE Evaluation
  1. **Treatment** **Schedule**

Study treatment will start within 30 days of eligibility determination.

Treatment will comprise 6 daily fractions of RT at 5 Gy per fraction followed

2-4 weeks later by approximately 4 months of chemotherapy.

Patients will be assessed for the primary outcome approximately 8 weeks (+/- 4 weeks) following completion of chemotherapy. Those who are not eligible for organ preservation will proceed with TME. All others will undergo organ preservation and surveillance for local regrowth for 2 years on protocol.

Treatment will be administered on an outpatient basis. No investigational or commercial agents or therapies other than those described may be administered with the intent to treat the patient's malignancy.

- - 1. **Radiation Therapy**
       1. **External Beam Equipment and General Techniques**

Linear accelerators with a minimum energy of 6-15 MV will be used. Patients will undergo CT treatment planning scans with patient supine or prone on a belly board per physician discretion and be treated in the same position. We will account for organ motion in multiple ways to ensure optimal target coverage while minimizing the dose to surrounding tissues, including: 1) cone-beam CT scans obtained on the linear accelerator in some cases to further verify target positioning, or 2) kilovoltage images through the On-Board Imaging system taken prior to delivery of each treatment field to verify position.

- - - 1. **Radiation Volumes and Prescribed Dose**

The intent of pre-operative treatment is to include the tumor bed plus the nodal groups at risk. A pelvic field will be treated initially using IMRT or 3D conformal radiotherapy (5 Gy x 5 fractions) with an additional optional boost fraction (5 Gy x 1 fraction) delivered sequentially to gross disease (including primary tumor and involved nodes). Assuming an alpha/beta ratio for rectal adenocarcinoma of approximately 5^63^, 30 Gy in 5 fractions has a biological equivalent dose of 60 Gy (conventional fractionation to 50.4 Gy in 28 fractions has a biological equivalent dose of 68.5 Gy). IMRT is recommended but not required.

Radiation simulation utilizing CT-based planning will be performed prior to RT. If possible, patients will be treated in the supine or prone position with a full bladder technique. IV contrast can be administered at physician’s discretion to patients without contrast allergy or compromised kidney function.

Gross tumor volume (GTV): This includes the primary tumor and any nodes believed to be involved grossly by metastatic disease. Assessment of the primary tumor and nodal disease may be made on the basis of procto/sigmoidoscopy, CT, PET-CT, MRI, and/or EUS. The entire rectal circumference at the level of the tumor should be included as GTV.

Clinical target volume 1 (CTV1): Includes the GTV, entire mesorectum and presacral space, and internal iliac nodes. The superior border will be placed at L5/S1 or L4/L5. If the tumor is a clinical T4 with anterior extension into an adjacent organ (i.e. prostate, cervix, bladder, vagina), the CTV is recommended to include the external iliac nodal regions. For patients with tumors invading the anal sphincter, the CTV is recommended to include the bilateral inguinal nodes and external iliac nodes.

Clinical target volume 2 (CTV2) – Includes the GTV plus 1-2 cm margin with additional inclusion of the entire mesorectum and presacral space up to physician discretion.

Planning target volume (PTV) – CTV1 and CTV2 will be expanded by a margin of 0.5 – 1 cm to account for daily setup variation to generate PTV1 and PTV2.

PTV1 will be treated to 25 Gy at 5 Gy/fraction.

PTV2 will be treated to 30 Gy at 5 Gy/fraction.

The sixth fraction to PTV2 may be omitted at the treating physician’s discretion due to patient anatomy and/or tumor location.

All treatment planning will be performed with computerized dosimetry using IMRT or 3D conformal radiotherapy. Plans will be normalized such that 95% of the planning target volume will receive the prescription dose. RT dose variation of +/- 12% to the GTV is acceptable.

**4.2.1.3.** **Radiation Dose Constraints**

- Small Bowel (contoured as bowel loops): 40 Gy Dmax, V30 < 5 cc, V33 < 1 cc
- Femoral head and neck (recommended): V15 < 50%; V20 < 35%
- Bladder: V25 < 50%
  - 1. **Chemotherapy**

Patients will be treated with approximately 4 months of FOLFOXIRI following RT. Patients who have performance status or conditions that may preclude use of FOLFOXIRI or mFOLFIRINOX may be treated with FOLFOX or XELOX instead at the discretion of the treating physician. **Modifications of dose and schedule are allowed as per clinical judgment of patient’s treating medical oncologist**. If deemed appropriate by the patient’s Stanford medical oncologist, the patient can receive chemotherapy treatment with their local oncologist.

- - - 1. **FOLFOXIRI**
- Oxaliplatin 85mg/m2 IV Day 1, dose rate per institutional standard of care
- Leucovorin 400 mg/m2 IV Day 1 (optional), dose rate per institutional standard of care
- Irinotecan 165mg/m2 IV Day 1, dose rate per institutional standard of care
- 5-Fluorouracil 3200mg/m2 IVI Day 1, over 48 hours
- 14-day cycle for 8 cycles
- If necessary to accommodate holidays, patient schedule or other justified circumstances, schedule may be modified by +/- 7 days
  - - 1. **mFOLFIRINOX**
- Oxaliplatin 85mg/m2 IV Day 1, dose rate per institutional standard of care
- Leucovorin 400 mg/m2 IV Day 1 (optional), dose rate per institutional standard of care
- Irinotecan 150mg/m2 IV Day 1, dose rate per institutional standard of care
- 5-Fluorouracil 2400mg/m2 IVI Day 1, over 46 hours
- 14-day cycle for 8 cycles
- If necessary to accommodate holidays, patient schedule or other justified circumstances, schedule may be modified by +/- 7 days
  - - 1. **FOLFOX**
- Oxaliplatin 85mg/m2 IV Day 1, dose rate per institutional standard of care
- Leucovorin 400 mg/m2 IV Day 1 (optional), dose rate per institutional standard of care
- 5-Fluorouracil 2400mg/m2 continuous infusion over 46 hours +/- 5-Fluorouracil 400mg/m2 IV bolus Day 1
- 14-day cycle for 8 cycles
- If necessary to accommodate holidays, patient schedule or other justified circumstances, schedule may be modified by +/- 7 days
  - - 1. **XELOX**
- Oxaliplatin 130 mg/m2 IV Day 1, dose rate per institutional standard of care
- Capecitabine 1000mg/m2 PO BID Days 1-14
- 21-day cycle for 6 cycles
- If necessary to accommodate holidays, patient schedule or other justified circumstances, schedule may be modified by +/- 7 days
  - 1. **Restaging during chemotherapy**

Patients will have restaging with clinical exam and MRI pelvis after 8 (+/-4) weeks of chemotherapy. If there is radiological or clinical evidence of disease progression, patient will be treated according to treating physician’s discretion. If a patient chooses to receive chemotherapy locally, they must return to Stanford for the clinical exam and other imaging procedures required for restaging.

- - 1. **Assessment of primary endpoint**

Patients’ responses to therapy will be evaluated clinically 8 (+/-4) weeks after completion of radiation and chemotherapy.

**4.2.5 Follow-Up Schedule (Time 0 = assessment of primary outcome)**

Patients who achieve cCR and are candidates for organ preservation per multidisciplinary review will undergo surveillance per protocol schedule. If tumor shows near complete response, patient may continue to be observed until the next evaluable timepoint. Additional scans and/or other tests may be performed and will be based on the clinical judgment of the treating physician.

Patients who are not candidates for organ preservation will proceed with TME. These patients will not proceed with the organ preservation surveillance schedule and instead be followed for cancer-specific outcomes (disease-free survival, colostomy-free survival and overall survival) per standard of care^64^ as directed by the treating physician. They will be assessed for AEs and health-related quality of life per Study Calendar.

For patients who achieve cCR and are on surveillance schedule, if suspicion of local regrowth (non-resolving residual abnormality that appears to increase in size or appears more suspicious in appearance or palpation, or imaging characteristics concerning for progression), patient will undergo a biopsy or a transanal full-thickness excision (TAE) if deemed appropriate to confirm. If biopsy is positive, patients will be managed per standard of care^64^ as directed by the treating physician.

Patients who pursue treatment for disease progression while on study will continue to be followed for OS, but discontinue routine AE assessment and QOLs, and follow-up with their treating physicians per standard of care.

After 2 years, all patients who initiated treatment on the protocol will be off study and followed for survival only. In general, patients who opt for organ preservation are recommended to continue to undergo surveillance years 3-5 as per below:

Recommend surveillance off study during years 3-5:

- Every 3-6 months
  - Exam and sigmoidoscopy
- Every 6 months
  - MRI pelvis and CT chest every 6 months

Follow-up PET/CTs or PET/MRI (preferred) are optional at any follow-up point, as per treating physician.

## General Concomitant Medication and Supportive Care Guidelines

## Supportive care

Patients should receive routine supportive care while on this study. This includes blood product support, antibiotic treatment, and treatment of other newly diagnosed or concurrent medical conditions.

## Antiemetics

Antiemetics may be used at the discretion of the attending physician.

## Blood products

## Blood products are permissible. The use of erythropoietin is strongly discouraged in the adjuvant setting and is not permitted in this trial.

## Neulasta and Neupogen

## Neulasta and Neupogen may be used to support maintenance of dose delivery and schedule according to physician discretion.

## Diarrhea

Diarrhea should be managed with loperamide. The recommended dose of loperamide is 4 mg at first onset, followed by 2 mg every 2-4 hours until diarrhea free (maximum 16 mg/day). Use of alternative agents such as Lomotil and tincture of opium is permissible.

## 4.4 Criteria for Removal from Study

Patients will receive treatment and follow-up as per Study Calendar until death or end of the study period, whichever occurs first.

A patient has the right to withdraw from the study at any time for any reason without

prejudice to his/her future medical care by the physician or at the institution, and the study PI has the right to withdraw a patient from study if they do not follow the treatment recommendations provided by the study investigators.

In the absence of treatment delays, treatment with study agents may span a period of approximately 4 months (including RT and chemotherapy). Patients may discontinue treatment for any of the following reasons:

- - Disease progression,
  - Intercurrent illness that prevents further administration of treatment,
  - Withdrawal due to patient non-compliance,
  - Withdrawal due to patient request,
  - Study termination, and/or
  - General or specific changes in the patient's condition which render the patient unsuitable for further treatment in the judgment of the investigator or treating physician.

If a patient does not return for a scheduled visit, every effort should be made to contact

him/her. In any such circumstance, every effort should be made to document the subject’s

outcome. Patients who leave Stanford or continue their care elsewhere will be contacted every 6 months by telephone, email, or mail. After three failed attempts to reach the patient, either via mail or telephone or a combination of the two, contact will cease and the patient will be considered lost to follow-up.

## 4.5 Alternatives

This study is optional. The alternative is to not participate in the study and receive standard of care treatment which includes TME.

# 5. INVESTIGATIONAL AGENT/DEVICE/PROCEDURE INFORMATION

# No investigational or commercial agents or therapies other than those described may be

# administered with the intent to treat the patient's malignancy.

# 6. DOSE MODIFICATIONS

# 6.1 Chemotherapy

FOLFOXIRI, mFOLFIRINOX, FOLFOX, XELOX, and 5-FU and capecitabine during radiation are standard regimens that have been widely used for the treatment of colon and rectal cancer for more than a decade. Investigators should refer to package and inserts and local pharmacy practices for a complete list of potential toxicities and adhere to best practices. Treatment interruptions and modifications should be made at the discretion of the treating physician.

Subjects should be assessed for Adverse Events (AEs) once during each chemotherapy cycle, and AEs will be recorded. Serious Adverse Events (SAEs) should be reported promptly as according to the guidelines in this protocol.

Chemotherapy infusion records should be kept in the patient’s study chart, either with the Medication Administration Record (MAR) or other clinic records which contain: Date of infusion, dose completed of each agent, modifications if any and reason for modification with supporting source documents as needed. Laboratory results prior to infusion should also be supplied in the patient study chart, as well as the consultation note if the patient is seen by the treating physician.

Below are recommendations and **not** requirements; **modifications of chemotherapy dose and schedule are allowed as per clinical judgment of patient’s treating medical oncologist.**

**6.1.1 Hematologic Toxicity**

- *For ANC 1,000/mm^3^–1,200/mm^3^:* Delay chemotherapy until ANC > 1,200*/mm^3^* 0then resume chemotherapy at the same dose level.
- *Second or More Occurrence of ANC 1000/mm^3^-1200/mm^3^*: Delay chemotherapy until ANC > 1,200*/mm^3^* then resume FOLFIRNOX with one dose level reduction for all subsequent cycles. **NOTE**: The dose of leucovorin is not reduced.
- *For ANC < 1,000/mm^3^:*  Delay chemotherapy until ANC > 1,200*/mm^3^*, then resume chemotherapy with one dose level reduction for all subsequent cycles. **NOTE**: The dose of leucovorin is not reduced.
- *For Febrile Neutropenia (defined as ANC < 1,000/mm^3^ and temperature ≥ 100.5°F):* Delay chemotherapy until resolution of fever and ANC > 1,200, then resume chemotherapy with one dose level reduction for all subsequent cycles. **NOTE**: The dose of leucovorin is not reduced.
- *For Platelets 50,000 K/ul – 75,000 K/ul:*  Delay chemotherapy until platelets > 75,000 then resume chemotherapy at the same dose level.
- *Second or More Occurrence of platelets 50,000 K/ul - 75,000 K/ul:* Delay chemotherapy until platelets > 75,000*K/ul*, then resume chemotherapy with one dose level reduction for all subsequent cycles. **NOTE**: The dose of leucovorin is not reduced.
- *For Platelets < 50,000 K/ul:*  Delay chemotherapy until recovery to Plts > 75,000 *K/ul* then resume chemotherapy with one dose level reduction for all subsequent cycles. **NOTE**: The dose of leucovorin is not reduced.
  - 1. **Non-Hematologic Toxicity**
       1. **Diarrhea**
- *For grade 2 Diarrhea (despite optimal medical management*):
- *First Occurrence*: Delay chemotherapy until recovery to grade ≤ 1 or baseline then resume chemotherapy at the same dose level.
- *Second or More Occurrence*: Delay chemotherapy until recovery to grade ≤ 1 or baseline, then resume chemotherapy with one dose level reduction for all subsequent cycles. **NOTE**: The dose of leucovorin is not reduced.
- *For grade 3 Diarrhea (despite optimal medical management):*
- *First Occurrence*: Delay chemotherapy until recovery to grade ≤ 1 or baseline, then resume 5-FU, leucovorin, and oxaliplatin, at the same dose level and irinotecan with one dose level reduction for all subsequent cycles.
- *Second or More Occurrence*: Delay chemotherapy until recovery to grade ≤ 1 or baseline, then resume chemotherapy with one dose level reduction for all subsequent cycles. **NOTE**: The dose of leucovorin is not reduced.
- *For grade 4 Diarrhea (despite optimal medical management):* Delay chemotherapy until recovery to grade ≤ 1 or baseline then resume chemotherapy with one dose level reduction for all subsequent cycles. **NOTE**: The dose of leucovorin is not reduced.
  - - 1. **Nausea/Vomiting**

The following dose modifications are based on toxicity experienced during a cycle.

- *For grade 3 Nausea/Vomiting (despite optimal medical management):*
- *First Occurrence*: Delay chemotherapy until recovery to grade ≤ 1 or baseline, then resume 5-FU and leucovorin, at the same dose level and oxaliplatin and irinotecan with one dose level reduction for all subsequent cycles.
- *Second or More Occurrence*: Delay chemotherapy until recovery to grade ≤ 1 or baseline, then resume chemotherapy with one dose level reduction for all subsequent cycles. **NOTE**: The dose of leucovorin is not reduced.
- *For grade 4 Nausea/Vomiting (despite optimal medical management):*

Delay chemotherapy until recovery to grade ≤ 1 or baseline, then resume chemotherapy with one dose level reduction for all subsequent cycles. **NOTE**: The dose of leucovorin is not reduced.

**6.1.2.3 Mucositis**

The following dose modifications are based on toxicity experienced at any time during a cycle.

- *For grade 3 Mucositis:*
- *First Occurrence*: Delay chemotherapy until recovery to grade ≤ 1, then *resume irinotecan, oxaliplatin, and leucovorin at the same dose level and 5-*FU with one dose level reduction for all subsequent cycles.
- *For Second or More Occurrence*: Delay chemotherapy until recovery to grade ≤ 1, then resume chemotherapy with one dose level reduction in irinotecan and oxaliplatin for all subsequent cycles. Dose of 5FU is reduced two dose levels for all subsequent cycles). **NOTE**: The dose of leucovorin is not reduced.
- *For grade 4 Mucositis:*

Delay chemotherapy until recovery to grade ≤ 1, then resume chemotherapy with one dose level reduction for all subsequent cycles. **NOTE**: The dose of leucovorin is not reduced.

**6.1.2.4 Peripheral Sensory Neuropathy**

- For paresthesia/dysethesia interfering with function and persisting between treatments: decrease oxaliplatin by one dose level for all subsequent cycles.
- For painful paresthesia/dysesthesia or symptoms that interfere with function and ADL, but improve (no longer painful or no longer interfering with ADL) between treatments: decrease oxaliplatin by one dose level for all subsequent cycles.
- For painful paresthesia/dysesthesia or symptoms that interfere with function and ADL that persists between treatments: discontinue oxaliplatin.
- For persistent disabling or life-threatening paresthesia/dysesthesia: discontinue oxaliplatin.
- For pharyngo-laryngeal dysesthesia: increase the duration of oxaliplatin infusion to 6 hours for subsequent cycles.

**6.1.2.5 Oxaliplatin-induced pharyngolaryngeal dysesthesias**

Should a patient develop oxaliplatin-induced pharyngolaryngeal dysesthesia, her/his oxygen saturation should be evaluated via a pulse oximeter; if normal, an anxiolytic agent may be given and the patient observed in the clinic until the episode has resolved. Following resolution of symptoms, patients may continue/resume oxaliplatin if the reaction is NOT determined to be an allergic reaction.

A table comparing pharyngo-laryngodysesthesia to platinum hypersensitivity reactions is presented below.

*Table 1 - Comparison of the Symptoms and Treatment of Pharyngolaryngodysesthesias and Platinum Hypersensitivity Reactions*

| **Clinical Symptoms** | **Pharyngo-Laryngeal Dysesthesias** | **Platinum Hypersensitivity** |
| --- | --- | --- |
| Dyspnea | Present | Present |
| Bronchospasm | Absent | Present |
| Laryngospasm | Absent | Present |
| Anxiety | Present | Present |
| O2 saturation | Normal | Decreased |
| Difficulty swallowing | Present (loss of sensation) | Absent |
| Pruritis | Absent | Present |
| Urticaria/rash | Absent | Present |
| Cold-induced symptoms | Yes | No |
| Blood pressure | Normal or increased | Normal or decreased |
| Treatment | Anxiolytics, observation in a controlled clinical setting until symptoms abate or at the physician’s discretion | Oxygen, steroids, epinephrine, broncho-dilators; fluids and vasopressors, if appropriate |

- - - 1. **Venous Thromboembolic Events**
- *For grade 2 or 3 venous thromboembolic event:* Continue chemotherapy at the same dose level. Do not use warfarin for therapeutic anticoagulation.
- *For grade 4 venous thromboembolic event:* Discontinue chemotherapy.

**6.1.2.7 Liver Function Tests**

- *For grade 2 Increased Blood Bilirubin:* Skip irinotecan until bilirubin improves to ≤ grade 1.
- For hyperbilirubinemia considered at least possibly related to irinotecan, then resume irinotecan with one dose level reduction for all subsequent cycles.
- For hyperbilirubinemia considered unrelated to irinotecan, resume irinotecan at the previous dose level.
- *For grade 3 or 4 Increased Blood Bilirubin:* Delay chemotherapy until bilirubin improves to ≤ grade 1. If bilirubin is thought to be due to a chemotherapy drug, then resume that drug at the next lower dose level and the other drugs at the same dose level when total bilirubin improves to ≤ grade 1.
- For hyperbilirubinemia considered at least possibly related to treatment (any drug) resume chemotherapy with one dose level reduction in suspect drug(s) for all subsequent cycles.
- For hyperbilirubinemia considered unrelated to treatment (all drugs), resume chemotherapy at the previous dose levels.
  - - 1. **Allergic Reactions**

*For grade 2 allergic reactions*: Interrupt infusion(s). Manage reaction according to institutional policy. Restart the infusion(s) when symptoms resolve to ≤ grade 1 and pre-treat before all subsequent doses.

*For grade 3 or Grade 4 allergic reactions:* Discontinue infusion. Manage reaction according to institutional policy. Discontinue chemotherapy.

- - - 1. **Other non-hematologic toxicities**

*For all other grade 3 non-hematologic toxicities considered at least possibly related to chemotherapy:*  Skip the responsible drug(s) until toxicity improves to ≤ grade 1, then resume the responsible drug(s) with one dose level reduction for all subsequent cycles.

*For grade 4 non-hematologic toxicities considered at least possibly related to chemotherapy:*  Discontinue the responsible drug(s).

# 6.2 Radiation Therapy

## Uninterrupted treatment is planned. RT will be held on any planned treatment day on which the patient exhibits ≥Grade 3 gastrointestinal toxicity that is reasonably attributed to pelvic RT. RT may be held until the toxicity resolves to less than Grade <2, at which point RT may be resumed. The patient will be assessed by a physician once during the course of RT during which any delays, interruptions, or discontinuation of treatment will be determined.

| **Symptom** | **CTCAE v 5.0**  **Criteria for Grading** | | | |
| --- | --- | --- | --- | --- |
|  | **1** | **2** | **3** | **4** |
|  |  |  |  |  |
| **Proctitis** | Rectal discomfort, intervention not indicated | Symptoms (e.g., rectal discomfort, passing blood or mucus), medical intervention indicated; limiting instrumental ADL | Severe symptoms; fecal urgency or stool incontinence; limiting self care ADL | Life-threatening consequences urgent intervention indicated |
| **Cystitis – noninfective** | Microscopic hematuria; minimal increase in frequency, urgency, dysuria, or nocturia; new onset of incontinence | Moderate hematuria; moderate increase in frequency, urgency, dysuria, nocturia or incontinence; urinary catheter placement or bladder irrigation indicated; limiting instrumental ADL | Gross hematuria; transfusion, IV medications or hospitalization indicated; elective endoscopic, radiologic or operative intervention indicated | Life-threatening consequences; urgent radiologic or operative intervention indicated |
| **Enterocolitis** | Asymptomatic; clinical or diagnostic observations only; intervention not indicated | Abdominal pain; mucus or blood in stool | Severe or persistent abdominal pain; fever; ileus; peritoneal signs | Life-threatening consequences; urgent intervention indicated |
| **Diarrhea** | Increase of <4 stools per day over baseline; mild increase in ostomy output compared to baseline | Increase of 4-6 stools per day over baseline; moderate increase in ostomy output compared to baseline | Increase of >=7stools per day over baseline; incontinence; hospitalization indicated; severe increase in ostomy output compared to baseline; limiting self care ADL | Life-threatening consequences; urgent intervention indicated |

# 7. ADVERSE EVENTS AND REPORTING PROCEDURES

The chemotherapies used on this trial are FDA-approved and are used as part of standard treatment in the US for patients with locally advanced rectal cancer. Therefore, all clinicians recruiting study subjects have considerable experience with this regimen. The toxicity of each specific agent is outlined below. The chemotherapy and radiation used in this clinical trial also represents the standard care for patients with locally advanced rectal cancer

## 7.1 Potential Adverse Events

## 7.1.1 Capecitabine

| **COMMON, SOME MAY BE SERIOUS**  In 100 people receiving Capecitabine, more than 20 and up to 100 may have: |
| --- |
| - Swelling of the body - Blisters on the skin - Redness, pain or peeling of palms and soles - Pain - Diarrhea, loss of appetite, nausea, vomiting - Sores in mouth which may cause difficulty swallowing - Anemia which may require blood transfusions - Infection, especially when white blood cell count is low - Bruising, bleeding - Feeling of "pins and needles" in arms and legs - Tiredness - Fever |

| **OCCASIONAL, SOME MAY BE SERIOUS**  In 100 people receiving Capecitabine, from 4 to 20 may have: |
| --- |
| - Blurred vision, dry or itchy eyes - Muscle spasms, body aches - Abnormal heartbeat - Restlessness, irritability - Swelling of face, fingers and lower legs - Constipation - Difficulty with balancing |

| **RARE, AND SERIOUS** In 100 people receiving Capecitabine, 3 or fewer may have: |
| --- |
| - Allergic reaction which may cause rash, low blood pressure, wheezing, shortness of breath, swelling of the face or throat - Difficulty speaking, walking or seeing - Internal bleeding which may cause blood in vomit or black tarry stools - Damage to the heart - A new cancer resulting from treatment of earlier cancer |

**7.1.2 5-Fluorouracil/Leucovorin**

| **COMMON, SOME MAY BE SERIOUS** In 100 people receiving 5-Fluorouracil, Leucovorin, more than 20 and up to 100 may have: |
| --- |
| - Hair loss - Redness, pain or peeling of palms and soles - Rash, increased risk of sunburn, itching - Diarrhea, nausea, vomiting, loss of appetite - Difficulty swallowing - Sores in mouth which may cause difficulty swallowing - Heartburn - Headache - Tiredness |

| **OCCASIONAL, SOME MAY BE SERIOUS** In 100 people receiving 5-Fluorouracil, Leucovorin, from 4 to 20 may have: |
| --- |
| - Chest pain - Blood clot - Belly pain - Internal bleeding which may cause black tarry stools - Infection, especially when white blood cell count is low - Anemia which may require blood transfusions - Cough, hoarseness - Bruising, bleeding - Allergic reaction which may cause rash, low blood pressure, wheezing, shortness of breath, swelling of the face or throat - Confusion - Abnormal eye movement, blurred vision, watering eyes - Discomfort from light - Swelling, redness, tingling and pain of hands and feet - Difficulty with balancing |

| **RARE, AND SERIOUS** In 100 people receiving 5-Fluorouracil, Leucovorin, 3 or fewer may have: |
| --- |
| - Damage to the heart which may cause shortness of breath - A new cancer resulting from treatment of earlier cancer |

**7.1.3. Oxaliplatin**

| **COMMON, SOME MAY BE SERIOUS** In 100 people receiving Oxaliplatin, more than 20 and up to 100 may have: |
| --- |
| - Anemia which may require blood transfusion - Diarrhea, nausea, vomiting, constipation, loss of appetite - Tiredness - Bruising, bleeding - Infection, especially when white blood cell count is low - Numbness, tingling or pain, "pins and needles" of the hands, feet, arms and legs - Tingling or a loss of feeling in your hands, feet, nose, or tightness in throat or jaw, or difficulty swallowing or breathing which may be made worse by exposure to cold - Pain - Fever, cough |

| **OCCASIONAL, SOME MAY BE SERIOUS** In 100 people receiving Oxaliplatin, from 4 to 20 may have: |
| --- |
| - Blood clot which may cause swelling, pain, or shortness of breath - Abnormal heartbeat which may cause fainting - Hearing loss - Problem with eyelid - Difficulty walking, using your hands, opening mouth, talking, with balance and hearing, smelling, eating, sleeping, emptying the bladder - Swelling of the body which may cause shortness of breath - Blockage of the airway which may cause shortness of breath, cough, wheezing - Bleeding from multiple sites including vaginal bleeding, bleeding of the testis, or bleeding of the brain - Internal bleeding which may cause black tarry stool, blood in vomit or urine, or coughing up blood - Sores in throat or mouth which may cause difficulty swallowing - Swelling and redness at the site of the medication injection - Liver damage which may cause yellowing of eyes and skin - Kidney damage which may require dialysis - Allergic reaction which may cause rash, low blood pressure, wheezing, shortness of breath, swelling of the face or throat - Weight gain, weight loss, dehydration - Dizziness, headache - Changes in taste, voice - Abnormal body movement including the eye and eyelid - Inability to move shoulder or turn head - Muscle weakness - Scarring of the lungs - Hair loss, itching, rash, hives |

| **RARE, AND SERIOUS** In 100 people receiving Oxaliplatin, 3 or fewer may have: |
| --- |
| - Redness, pain or peeling of palms and soles - A new cancer resulting from treatment of earlier cancer |

**7.1.4 Irinotecan**

| **COMMON, SOME MAY BE SERIOUS**  In 100 people receiving Irinotecan, more than 20 and up to 100 may have: |
| --- |
| - Severe diarrhea - Constipation, nausea, vomiting - Infection, especially when white blood cell count is low - Hair loss - Loss of appetite, weight loss - Anemia which may cause tiredness, or may require a blood transfusion - Fever, pain - Dizziness, tiredness, weakness - Cough, shortness of breath - Sores in mouth - Rash - Bruising, bleeding |

| **OCCASIONAL, SOME MAY BE SERIOUS**  In 100 people receiving Irinotecan, from 4 to 20 may have: |
| --- |
| - Allergic reaction which may cause rash, low blood pressure, wheezing, shortness of breath, swelling of the face or throat - Blood clot which may cause swelling, pain, shortness of breath - Scarring of the lungs |

| **RARE, AND SERIOUS**  In 100 people receiving Irinotecan, 3 or fewer may have: |
| --- |
| - A new cancer resulting from treatment of earlier cancer |

**7.1.5 Radiation Therapy**

Expected acute side effects of radiation therapy include:

- - Abdominal cramping
  - Diarrhea
  - Skin erythema
  - Dysuria
  - Tenesmus
  - Leukopenia
  - Lymphopenia
  - Thrombocytopenia

Late effects of radiation therapy include:

- - Increased frequency of bowel movements
  - Bowel urgency
  - Infertility in women who have not undergone an ovarian transposition
  - Early menopause in pre-menopausal women who have not undergone an ovarian transposition
  - Vaginal dryness and narrowing

## 7.1.6 Surgical complications

Surgery is part of the standard treatment of most patients with rectal cancer. The most

common complications of surgery for rectal cancer include wound infection, pelvic infection, bleeding, bowel obstruction, vein thrombosis, pneumonia, cardiac arrhythmia, and heart attack.

Long-term side effects include frequent bowel movements, urinary problems, sexual dysfunction, and hernia.

Surgical difficulty will be graded using a 1-10 scale for pelvic fibrosis and technical difficulty^20^.

## 7.2 Adverse Event Reporting

As Coordinating Center, we will follow guidelines from Stanford’s Research Compliance Office and Cancer Clinical Trials Office (CCTO) for defining, identifying, and reporting events as defined below.

**7.2.1** **Serious Adverse Events (SAEs)**

A Serious Adverse Event (SAE) is defined as: Any adverse event that meets any of the following criteria:

- Fatal (i.e., the adverse event actually causes or leads to death)
- Life threatening (i.e., the adverse event, in the view of the investigator, places the patient at immediate risk of death). This does not include any adverse event that had it occurred in a more severe form or was allowed to continue might have caused death
- Requires or prolongs inpatient hospitalization
- Results in persistent or significant disability/incapacity (i.e., the adverse event results in substantial disruption of the patient’s ability to conduct normal life functions)
- Congenital anomaly/birth defect in a neonate/infant born to a mother exposed to study drug
- Significant medical event in the investigator's judgment (e.g., may jeopardize the patient or may require medical/surgical intervention to prevent one of the outcomes listed above)

**7.2.1.1** **Reporting SAEs**

- SAEs should be graded according to the NCI Common Terminology Criteria for Adverse Events, Version 5.0. NCI CTCAE v5 can be downloaded from <http://ctep.cancer.gov/protocolDevelopment/electronic_applications/ctc.htm>”

SAEs Grade 3 and above require prompt reporting. Routine SAE Grade 3+ reporting will be to [CCTO-Safety@stanford.edu](mailto:CCTO-Safety@stanford.edu). These reports will also be sent to Dr. Erqi Pollom and Stanford Research Staff (Research Nurse or Research Coordinators) with subject line: “SECURE: SHORT FOX STUDY Serious Adverse Event.” A Case Report Form (CRF) describing the event must be supplied and can be sent as an attachment to the email. It could be generated from the OnCore entry (as a pdf or report).

Stanford research staff will notify Stanford IRB via ‘eProtocol’ as per regulatory and institutional guidelines. The SAE may also be forwarded to other Stanford regulatory boards, e.g., DSMC, as applicable.

**7.2.2 Unanticipated Problems (UPs)**

Per Stanford IRB, UPs are events involving risks to participants or others and must meet ALL 3 criteria below:

**1. Unexpected**: in terms of nature, severity, or frequency, given (a) the research procedures described in the protocol-related documents, and (b) the characteristics of the subject population being studies; AND

**2. Related** to participation in the research: or there is a reasonable possibility that the incident, experience, or outcome may have been caused by the procedures involved in the research; or if a device is involved, probably caused by, or associated with the device; AND

**3. Harmful**: suggests that the research places subjects or others at a greater risk of harm (including physical, psychological, economic, or social harm) than was previously known or recognized.

UPs *generally will warrant consideration of substantive changes in the research protocol or informed consent process/document, or other corrective actions, in order to protect the safety, welfare, or rights of subjects or others. Due to this, UPs will be reported promptly to Stanford IRB following the below guidelines.*

A UP may also be an AE or SAE and can be noted in OnCore.

**7.2.2.1 Reporting UPs**

UPs should be entered into OnCore and reported within 24 hours of learning of the event. Reporting can be done via email to Stanford Cancer Clinical Trials Office (CCTO) at [ccto-safety@stanford.edu](mailto:ccto-safety@stanford.edu), copying Dr. Erqi Pollom and Stanford research staff (Research Nurse or Research Coordinators), with subject line: “SECURE: SHORT FOX STUDY Unanticipated Problem.” A Case Report Form (CRF) describing the event must be supplied and can be sent as an attachment to the email. It could be generated from the OnCore entry (as a pdf or report).

Stanford research staff will notify Stanford IRB via ‘eProtocol’ as per regulatory guidelines. The UP may also be forwarded to other Stanford regulatory boards, e.g., DSMC, as applicable.

| SAE and UP Reporting Requirements | | | | |
| --- | --- | --- | --- | --- |
| Hospitalization | Grade 1 | Grade 2 | Grade 3 | Grade 4/5 |
| Hospitalization≤ 24 hrs | UP's within 24 hours | UP's within 24 hours | Report SAE promptly, and UP within 24 hours | Report SAE promptly, and UP within 24 hours |
| Hospitalization≥ 24 hrs | UP's within 24 hours | UP's within 24 hours | Report SAE promptly, and UP within 24 hours | Report SAE promptly, and UP within 24 hours |
| **SAE Definition**: Fatal, life threatening, requires hospitalization or prolongs hospitalization, results in persistent or significant disability, causes congenital anomaly/birth defect or significant medical event. | | | | |
| **Unexpected Problem (UP) Definition**: Unexpected in terms of nature, severity, or frequency. Related to participation in the research. And Harmful suggesting that the research places subjects or others at a greater risk of harm. | | | | |
| **To report all UP's and SAEs Grade 3 and above**: 1) Report to Stanford IRB via eProtocol 2) Enter the event into OnCore, 3) send email to Dr. Erqi Pollom and Clinical Trial Manager with the subject line: "SECURE: SHORT FOX STUDY Serious Adverse Event" and 4) Include Case Report Form describing the event and submit follow up reports for the resolution of the event. For any questions regarding whether an event is an SAE or a UP, please email Protocol Director and Clinical Trial Manager. | | | | |
| **(Protocol Director) Dr. Erqi Pollom:** [erqiliu@stanford.edu](mailto:erqiliu@stanford.edu)  **Clinical Trial Manager:** [**rachelf@stanford.edu**](mailto:rachelf@stanford.edu) | | | | |

**7.2.3. Adverse Events (AEs) and AE Monitoring**

An AE is defined as any untoward medical occurrence in a clinical investigation subject, regardless of causal attribution.

All AEs should be graded according to the NCI Common Terminology Criteria for Adverse Events, Version 5.0, available at <http://ctep.cancer.gov/reporting/ctc.html>.

AEs should be:

- Assessed at each consultation or follow-up visit,
- Assessed during each chemotherapy cycle (prior to infusion is acceptable), and
- Recorded in patient’s medical chart using a Smartphase

Laboratory tests (CBC with differential and CMP) should also be obtained with each chemotherapy administration, and abnormal values also noted indicating AE Grade if applicable. For those patients who choose to receive chemotherapy locally, they will have a televisit, over a secure server, before every cycle where their Stanford treating physician will review and assess any adverse events.

AEs should also be:

- Reported to Stanford as per protocol guideline and upon request

- Noted in patient study charts and binder

- Entered into REDCap

AEs in REDCap should remain current and may be requested by Stanford for study renewals, audits, reports, analyses or other submissions for Stanford IRB, SRC, or DSMC. AEs may also be reviewed by members of the study monitoring group in meetings, audits, and/or site visits.

Updates and outcomes of AEs, SAEs, UPs, and deviations may take place during teleconferences or more frequently as needed.

**7.2.4 Deviations**

A protocol deviation is any unapproved discrepancy from a protocol research plan or Good Clinical Practice (GCP) guidelines, except where necessary to eliminate an immediate hazard to trial subjects.

**7.2.4.1 Documenting and Reporting Deviations**

**Serious or Major Deviations**

Research staff should promptly report serious or major deviations, or those that affect participant eligibility, informed consent, or protocol endpoints; and/or any deviation that could potentially result in harm to participants. These should be reported within 24 hours of the deviation or of learning of the deviation, by submitting as necessary to the Stanford IRB via ‘eProtocol’, and then entering them into OnCore. The OnCore record should include the following:

- description of the deviation,
- corrective action taken,
- a statement whether the patient was harmed or could have been potentially harmed by the deviation, and
- whether the deviation was reported to the Stanford IRB.

Stanford research staff should also be immediately notified of the deviation, via email to Dr. Erqi Pollom, copying the study Research Nurse or Research Coordinator with subject line: “SECURE: SHORT FOX STUDY Major Deviation.” A Case Report Form (CRF) describing the event is required and could be generated from the OnCore entry (as a pdf or report) and attached to the email.

Stanford DSMC reviews deviations in OnCore to ensure completeness of the deviation report and ensure the safety of trial participants. The DSMC may request corrective action and/or prompt reporting to Stanford IRB. If reporting to Stanford IRB is required, Stanford’s research staff (Study Nurse or Coordinator) will assist with this by entering the deviation in ‘eProtocol’.

**Minor Deviations**

Deviations not meeting the above criteria for major or ‘serious,’ or that do not have a significant effect on the subject’s rights, safety, or welfare, or on the integrity of the data -- e.g., conducting a protocol-required visit out of the protocol ‘window’ -- should be documented on the Deviation Log in Appendix V.

Deviations in OnCore or in Deviation logs should remain current at all time, and may be requested by Stanford for study renewals, audits, reports, analyses or other submissions for Stanford IRB, SRC, or DSMC.

# 8. CORRELATIVE/SPECIAL STUDIES

## 8.1 ctDNA study

**8.1.1 Collection of Specimen**

Blood will be drawn when patient is enrolled and registered on-study and during treatment for all patients and then at each follow-up along with the patient’s clinical labs among patients who pursue organ preservation (see Study Calendar). Tumor tissue and germline DNA will be sequenced to identify mutations to track in ctDNA blood samples. Therefore, whenever possible, tumor tissue will be collected, consisting of a punch from a paraffin embedded tissue block or several unstained slides. Samples will be sent to the Diehn lab at Stanford for processing and analysis. Possible timepoints for requesting tumor tissue include: patient’s biopsy from time of diagnosis (this would take place retroactively), surgical resection specimens, and any additional biopsies obtained while on study.

**8.1.2 Shipping of Specimen**

N/A. Samples will be analyzed on-site at Stanford. In the event that the patient undergoes a biopsy at an outside institution, the research team may request that the outside site ship a tissue sample to Stanford for processing and analysis.

**8.1.3 Site Performing Correlative Studies**

ctDNA analyses will be performed on the collected blood specimens retrospectively at Stanford in the laboratory of Dr. Maximillian Diehn by his research staff using research-use only platforms (CAPP-Seq and PhasED-Seq). These analyses will NOT be used for eligibility or to inform treatment decisions and is for “after the fact” research analysis only.

**8.1.4 Coding of specimens for privacy protection**

At the time of enrollment each patient will be given a specific confidential identification number (IDN). Specimens will be stored under the patient’s IDN. The information can be shared with other investigators listed on this protocol. Study data will be maintained in password protected computer files (protected online database). Only research personnel will have access to this information.

# 9. STUDY CALENDAR

| Activity | Pre-study (to be performed within 45 days of enrollment unless otherwise specified) | RT | Chemo | Primary Outcome Assessment 8 (+/- 4) weeks after Chemo (Time 0) | Follow-up after Time 0^g^ | | |
| --- | --- | --- | --- | --- | --- | --- | --- |
|  |  |  |  |  | Every 3 months | Months 3, 6, 12, 18, 24 | Months 6, 12, 18, 24 |
| Pregnancy Test^a^ | X |  |  |  |  |  |  |
| Biopsy within 180 days of enrollment | X |  |  | X^e^ | X^e^ |  |  |
| Clinical Exam with DRE^b^ | X |  | X^d^ | X | X |  |  |
| Procto/Sigmoidoscopy within 90 days of enrollment | X |  | X^d^ | X | X |  |  |
| CT chest/abdomen/pelvis^c^ | X |  | X^d^ | X |  |  | X |
| MRI pelvis | X |  | X^d^ | X |  | X |  |
| Labs-CBCD, CMP, CEA | X |  | X^i^ | X |  |  | X |
| ECOG | X |  |  |  |  |  |  |
| HRQOL | X^j^ |  |  | X |  |  | X^f^ |
| PET/MRI or FDG-PET^c^ | X |  |  | X |  |  |  |
| AE evaluation | X^j^ | X | X | X^L^ | X^f^ |  |  |
| ctDNA | X^j^ | X^k^ |  | X |  | X^h^ |  |

^a^Negative pregnancy test by urine, for women non-post-menopausal as defined in Appendix.

^b^DRE may be deferred for clinical reasons, as determined by treating physician.

^c^MRI abdomen can substitute for CT abdomen/pelvis per physician discretion; PET imaging is *optional*. PET/MRI is preferred.

^d^Restaging after 8 (+/-4) weeks of chemotherapy. Procto/sigmoidoscopy and CT CAP can be omitted per physician discretion.

^e^ If biopsy is needed to rule out residual or progressive tumor, per the discretion of the surgeon

^f^After TME, patients will leave surveillance schedule but continue to receive HRQOLs, and be assessed for treatment-related AEs

^g^Follow-up appointments and activities 2 weeks before and 2 weeks after each timepoint are permitted, and may be completed virtually.

^h^Among only those managed with organ preservation

^i^CEA does not need to be collected; pre-study CBCD, CMP may suffice for cycle 1 Day 1 chemo labs

^j^Activities to be done after enrollment and before treatment begins

^K^ctDNA will be collected following completion of RT

# ^L^An additional AE evaluation may be scheduled up to 3 months after competition of chemotherapy if needed

# 10. MEASUREMENTS

**For clinicaltrials.gov and Stanford Clinical Trials Directory compliance**

Primary Outcome Measure Definition: Proportion of patients who achieve a clinical complete response following therapy, expressed as a number and proportion without dispersion.

- Title: clinical complete response
- Time Frame: 8 (+/-4) weeks after completion of RT and chemotherapy
- Safety Issue: Is this outcome measure assessing a safety issue? No

## 10.1 Primary Outcome Measure

The primary outcome will be assessed 8 (+/- 4) weeks following completion of RT and chemotherapy. A cCR requires multidisciplinary review and is defined as follows: 1) No residual tumor or suspicious lymph nodes on MRI (Table 1, 2), 2) no residual tumor on direct visualization with endoscopy OR a negative biopsy of any residual or suspicious ulcer or mucosal abnormality and 3) no palpable tumor on DRE (if applicable).

Table 1. Magnetic resonance imaging tumor regression grade (mrTRG)^51^

| **mrTRG 1** | Complete radiological response (linear scar only) |
| --- | --- |
| **mrTRG 2** | Good response (dense fibrosis, no obvious tumor signal) |
| **mrTRG 3** | Moderate response (>50% fibrosis and visible intermediate signal) |
| **mrTRG 4** | Slight response (mostly tumor) |
| **mrTRG 5** | No response/regrowth of tumor |

##

Table 2. Malignant Morphologic Criteria and Lymph Node Size^56^

– indicates not suspicious for malignancy; + indicates suspicious for malignancy

| Number of malignant morphologic criteria* | Lymph Node Size** | | |
| --- | --- | --- | --- |
|  | <5mm | 5-9 mm | >9 mm |
| None | - | - | + |
| Two | - | + | + |
| Three | + | + | + |

*Irregular borders, heterogenous signal intensity, round shape

**Measured in largest short axis.

## 10.2 Secondary Outcome Measures

- Toxicity at least possibly attributed to treatment, graded using CTCAE v5 criteria and assessed up to 3 months after completion of RT and chemotherapy, expressed as a number and proportion without dispersion.
- 2-year local regrowth rate where local regrowth is defined as the presence of adenocarcinoma within the rectal wall or within the mesorectum confirmed by pathology, expressed as a percentage with its 95% confidence interval.
- DFS defined as the time from the start date of RT to the date of the first documented progression or death due to any cause assessed up to 2 years after completion of chemotherapy. Patients will be censored at the last date of follow-up if lost to follow-up prior to two years, expressed as a median with interquartile range.
- Colostomy-free survival defined as the time from the start date of RT to the date of colostomy or death due to any cause assessed up to 2 years after completion of chemotherapy, expressed as a median with interquartile range. Patients will be censored at the last date of follow-up if lost to follow-up prior to two years.
- OS defined as death from any cause from start date of RT until death, study completion, or loss to follow-up, whichever occurs first. This will be reported as median survival time with interquartile range.

**10.3 Exploratory Outcome Measures**

- HRQOL in Appendices (B-H) will be collected by paper or electronically by the research coordinator at every study assessment visit or via mail or the myHealth messaging system (see Study Calendar). Data will be collected and stored in RedCap. Patients will receive 5 surveys (tailored to patient gender and the presence of a stoma). The entire functional assessment can be completed in less than 30 minutes, with an average of 15-20 minutes per patient.
  - Bowel function index (BFI)  ^65^
    - We will use the 19-item BFI to assess bowel function. The psychometric properties of the instrument have been previously published and all subscales and total score show good internal consistency.
  - Low Anterior Resection Score (LARS)  ^66.67^
    - All patients without a stoma will also complete 5 additional questions from the LARS. The is a validated 5 question index that was specifically developed to categorize bowel dysfunction after low anterior resection. In the LARS Score patients are asked to describe symptoms which best describe daily life. There is no specific time period, but at most the averages are over the past week. The LARS score has a range of 0-42 with 0-20 being No LARS, 21-29 being Minor LARS, and 30-42 being Major LARS.
  - Stoma QOL ^68^
    - We will also use the 21-item Stoma QOL for patients undergoing APR.
  - Sexual function
    - To assess sexual function, we will use gender-specific questionnaires: 5-item International Index of Erectile Function (IIEF-5) and 6-item Female Sexual Functioning Index^69-71^ .
  - Bladder function
    - Bladder function will be assessed using the International Prostate Symptom Score^72^ .
  - EuroQOL Group five dimensions Health Questionnaire (EQ-5D)^73^
    - The EQ-5D is a widely used descriptive system of HRQOL states. It consists of five dimensions (mobility, self-care, usual activities, pain/discomfort, anxiety/depression) each of which can take one of five responses. The responses record five levels of severity (no problems/slight problems/moderate problems/severe problems/extreme problems or unable to function) within a particular EQ5D dimension. The EQ5D is almost always combined with a simple linear analogue scale that asks respondents to rate their overall health on a vertical scale of 0 to 100.

# 11. REGULATORY CONSIDERATIONS

## 11.1 Institutional Review of Protocol

The protocol, the proposed informed consent and all forms of participant information related to the study (e.g. advertisements used to recruit participants) will be reviewed and approved by the Stanford IRB and Stanford Cancer Institute Scientific Review Committee (SRC). Any changes made to the protocol will be submitted as a modification and will be approved by the IRB prior to implementation. The Protocol Director will disseminate the protocol amendment information to all participating investigators.

## 11.2 Data and Safety Monitoring Plan

The Stanford Cancer Institute Data and Safety Monitoring Committee (DSMC) will be the monitoring entity for this study. The DSMC will audit study-related activities to determine whether the study has been conducted in accordance with the protocol, local standard operating procedures, FDA regulations, and Good Clinical Practice (GCP).  This may include review of the following types of documents participating in the study: regulatory binders, case report forms, eligibility checklists, and source documents. In addition, the DSMC will regularly review serious adverse events and protocol deviations associated with the research to ensure the protection of human subjects. Results of the DSMC audit will be communicated to the IRB and the appropriate regulatory authorities at the time of continuing review, or in an expedited fashion, as needed.

## 11.3 Data Management Plan

The Protocol Director, or his/her designee, will prepare and maintain adequate and accurate participant case histories with observations and data pertinent to the study. Study specific Case Report Forms (CRFs) will document treatment outcomes for data analysis. Case report forms will be developed using the OnCore and RedCap database system and will be maintained by the study coordinator. CRFs will be kept in a locked office, only accessible to the research team. The Investigator will certify that the data entered are complete and accurate.

Study data will be maintained in password protected databases, computer, and/or electronic files. Only research personnel listed on this protocol will have access to this information. Only the patients unique IDN will be used. Specimens will be stored under the patient’s IDN. The patient’s name or other public identifiers will not be included in any information shared with other investigators. Survey responses, as well as name and date of birth, will be obtained via the secure, web-based program RedCap that is PHI approved by the Stanford IRT Department. This will be password protected and only accessible by the study coordinators and PI.

# 12. STATISTICAL CONSIDERATIONS

## 12.1 Statistical Design

The primary objective of this study is to evaluate the efficacy of SCRT and FOLFOXIRI for rectal cancer as measured by cCR. We plan to use an optimal Simon 2 stage design to meet our primary objective. In addition, we plan to assess toxicity at the first stage.

## 12.2 Interim analyses

We plan to conduct one interim analysis for futility. We will assess efficacy and toxicity during this analysis. Following an optimal Simon 2 stage design for efficacy, we will enroll 17 patients and assess cCR. If less than or equal to 3 patients have cCR we will stop the study for futility. Otherwise, we will continue the study and enroll 20 more patients. At the end of the study, if 11 or more patients have cCR, we will deem this treatment strategy acceptable.

We assume an acceptable toxicity rate of ≤ 25% with an unacceptable toxicity rate of 40%. Thus, at the interim analysis, if 7 or more patients have toxicity (defined as non-hematologic CTCAE v5 grade 4 or higher toxicity at least possibly related to treatment), we will stop the study and consider the treatment strategy unsafe. Otherwise, we will continue the study and conclude that the treatment is acceptable if 13 or fewer patients of the 37 total patients have toxicities. Assuming that the true toxicity rate is 25%, the probability of stopping the study at the interim analysis for toxicity is 11% and for rejecting the null hypothesis is 13%. If the true toxicity rate is 40%, the probability of stopping the study early is 55% and probability of rejecting the null hypothesis is 74%.

## 12.3 Descriptive Statistics and Exploratory Data Analysis

Demographic and other baseline data including disease characteristics will be listed and summarized. Categorical data will be presented as frequencies and percentages. For continuous data, mean, standard deviation, median, 25th and 75th percentiles will be presented.

## 12.4 Primary Analysis

- - 1. **Analysis Population**

The analysis population will include all patients who initiated the RT and chemotherapy regimen (intent-to-treat). Patients who withdraw from the study prior to start of treatment will be replaced.

- - 1. **Analysis Plan**

Our primary analysis will be the calculation of the cCR rate, defined as the percentage of patients who achieved cCR in the analysis population. The cCR and the two-sided 95% Clopper-Pearson (exact) confidence interval will be calculated for the analysis population.

**12.5 Secondary Analysis**

**12.5.1 Analysis Population**

For the secondary outcome of local regrowth rate, the analysis population will include all patients managed with organ preservation approach. For the secondary outcomes of toxicity, DFS, colostomy-free survival, OS and HRQOL, the analysis population will be the same set of patients included for the primary analysis.

**12.5.2 Analysis Plan**

For the toxicity outcome, we will calculate the proportion of patients meeting our definition of toxicity. In addition, we will present the frequency and percent of other adverse events reported in the study.

Local regrowth rate will be calculated as the proportion of patients with local regrowth based on the secondary analysis population. The local regrowth rate and its 95% confidence interval will be calculated.

Disease-free survival is defined as the time from start of RT therapy to date of progression or death due to any cause. If a subject is not known to have progressed or died, then DFS will be censored at the latest date the subject was known to be alive or disease-free (on or before the cut-off date). The DFS distribution will be estimated using the Kaplan-Meier method, and the Kaplan-Meier curves, medians and 95% confidence intervals of the medians will be presented.

Colostomy-free survival distribution will be estimated using the Kaplan-Meier method, and the Kaplan-Meier curves, medians and 95% confidence intervals of the medians will be presented.

OS is defined as the time from start of RT therapy to date of death due to any cause. If a subject is not known to have died, then OS will be censored at the latest date the subject was known to be alive (on or before the study end date). The OS distribution will be estimated using the Kaplan-Meier method, and the Kaplan-Meier curves, medians and 95% confidence intervals of the medians will be presented.

For the HRQOL measures, we will calculate the mean and standard deviations of each of the individual indices at baseline and every 3 months during the duration of the study; we will graphically depict scores at each time point. In addition, we will calculate the difference of the QoL scores at the baseline, time of cCR, and last follow up date and report summary statistics.

## 12.6 Sample Size

**12.6.1 Accrual estimates**

We plan to enroll patients from radiation oncology, medical oncology, surgical oncology clinics as well as the GI multidisciplinary tumor board from 2020 to 2025. We estimate that 6 patients per month will be eligible for this study and that we will accrue 0-1 patients each month. We will allow patients to receive chemotherapy locally as prescribed by the trial and if necessary, can consider opening the trial at our satellite facilities to maximize our accrual numbers. If we do not meet our expected accrual rate, we will extend the study period and consider opening this trial at other institutions.

**12.6.2 Sample size justification**

We plan to use a Simon 2 stage design to address our primary objective. Assuming a one-sided type one error of 0.1, power of 0.9, a null cCR of 0.2 versus an alternate cCR of 0.4, we plan to enroll a total of 37 patients. After 17 patients have been enrolled, we will assess the number of patients with cCR to determine whether to stop the study for futility or to continue. If less than or equal to 3 patients have cCR the study will be stopped. Otherwise, we will continue to enroll 20 more patients. The null hypothesis will be rejected if 11 or more total patients have cCR.

**12.6.3 Effect size justification**

The historical rate for cCR based on previous literature is estimated to be ~20% based on data from randomized trials^33,43,50^ and systematic review and pooled analysis of 17 studies comprising 692 patients treated with a variety of neoadjuvant CRT approaches^74^ .

## 12.7 Criteria for future studies

Based on our criteria stated above, if the study is not stopped at the interim analysis, if 11 or more patients of the 37 patients have cCR and 13 or fewer patients have grade 4+ non-hematologic toxicity, we will deem this treatment strategy acceptable.

# 13. REFERENCES

1. Siegel RL, Miller KD, Jemal A. Cancer statistics, 2019. *CA Cancer J Clin*. 2019;69(1):7-34. doi:10.3322/caac.21551

2. Gunderson LL, Sosin H. Areas of failure found at reoperation (second or symptomatic look) following “curative surgery” for adenocarcinoma of the rectum. Clinicopathologic correlation and implications for adjuvant therapy. *Cancer*. 1974;34(4):1278-1292. doi:10.1002/1097-0142(197410)34:4<1278::aid-cncr2820340440>3.0.co;2-f

3. Yamada K, Ishizawa T, Niwa K, Chuman Y, Akiba S, Aikou T. Patterns of pelvic invasion are prognostic in the treatment of locally recurrent rectal cancer. *Br J Surg*. 2001;88(7):988-993. doi:10.1046/j.0007-1323.2001.01811.x

4. Sauer R, Rödel C, Martus P, Hess CF, Schmidberger H. Preoperative versus Postoperative Chemoradiotherapy for Rectal Cancer. *N Engl J Med*. 2004:10.

5. Ngan SY, Burmeister B, Fisher RJ, et al. Randomized trial of short-course radiotherapy versus long-course chemoradiation comparing rates of local recurrence in patients with T3 rectal cancer: Trans-Tasman Radiation Oncology Group trial 01.04. *J Clin Oncol Off J Am Soc Clin Oncol*. 2012;30(31):3827-3833. doi:10.1200/JCO.2012.42.9597

6. Li J, Li L, Yang L, et al. Wait-and-see treatment strategies for rectal cancer patients with clinical complete response after neoadjuvant chemoradiotherapy: a systematic review and meta-analysis. *Oncotarget*. 2016;7(28):44857-44870. doi:10.18632/oncotarget.8622

7. Sebag-Montefiore D, Stephens RJ, Steele R, et al. Preoperative radiotherapy versus selective postoperative chemoradiotherapy in patients with rectal cancer (MRC CR07 and NCIC-CTG C016): a multicentre, randomised trial. *The Lancet*. 2009;373(9666):811-820. doi:10.1016/S0140-6736(09)60484-0

8. Smith JJ, Strombom P, Chow OS, et al. Assessment of a Watch-and-Wait Strategy for Rectal Cancer in Patients With a Complete Response After Neoadjuvant Therapy. *JAMA Oncol*. January 2019:e185896. doi:10.1001/jamaoncol.2018.5896

9. Russell MM, Ganz PA, Lopa S, et al. Comparative Effectiveness of Sphincter-Sparing Surgery Versus Abdominoperineal Resection in Rectal Cancer: Patient-Reported Outcomes in National Surgical Adjuvant Breast and Bowel Project Randomized Trial R-04. *Ann Surg*. 2015;261(1):144-148. doi:10.1097/SLA.0000000000000594

10. Valk MJM van der, Hilling DE, Bastiaannet E, et al. Long-term outcomes of clinical complete responders after neoadjuvant treatment for rectal cancer in the International Watch & Wait Database (IWWD): an international multicentre registry study. *The Lancet*. 2018;391(10139):2537-2545. doi:10.1016/S0140-6736(18)31078-X

11. Smith JD, Ruby JA, Goodman KA, et al. Nonoperative Management of Rectal Cancer With Complete Clinical Response After Neoadjuvant Therapy: *Ann Surg*. 2012;256(6):965-972. doi:10.1097/SLA.0b013e3182759f1c

12. Renehan AG, Malcomson L, Emsley R, et al. Watch-and-wait approach versus surgical resection after chemoradiotherapy for patients with rectal cancer (the OnCoRe project): a propensity-score matched cohort analysis. *Lancet Oncol*. 2016;17(2):174-183. doi:10.1016/S1470-2045(15)00467-2

13. Martens MH, Maas M, Heijnen LA, et al. Long-term Outcome of an Organ Preservation Program After Neoadjuvant Treatment for Rectal Cancer. *J Natl Cancer Inst*. 2016;108(12):djw171. doi:10.1093/jnci/djw171

14. Maas M, Beets-Tan RGH, Lambregts DMJ, et al. Wait-and-See Policy for Clinical Complete Responders After Chemoradiation for Rectal Cancer. *J Clin Oncol*. 2011;29(35):4633-4640. doi:10.1200/JCO.2011.37.7176

15. Habr-Gama A, Gama-Rodrigues J, São Julião GP, et al. Local Recurrence After Complete Clinical Response and Watch and Wait in Rectal Cancer After Neoadjuvant Chemoradiation: Impact of Salvage Therapy on Local Disease Control. *Int J Radiat Oncol*. 2014;88(4):822-828. doi:10.1016/j.ijrobp.2013.12.012

16. Habrgama A, Perez R, Proscurshim I, et al. Patterns of Failure and Survival for Nonoperative Treatment of Stage c0 Distal Rectal Cancer Following Neoadjuvant Chemoradiation Therapy. *J Gastrointest Surg*. 2006;10(10):1319-1329. doi:10.1016/j.gassur.2006.09.005

17. Appelt AL, Pløen J, Harling H, et al. High-dose chemoradiotherapy and watchful waiting for distal rectal cancer: a prospective observational study. *Lancet Oncol*. 2015;16(8):919-927. doi:10.1016/S1470-2045(15)00120-5

18. Habr-Gama A, Perez RO, Nadalin W, et al. Operative Versus Nonoperative Treatment for Stage 0 Distal Rectal Cancer Following Chemoradiation Therapy. *Ann Surg*. 2004;240(4):711-718. doi:10.1097/01.sla.0000141194.27992.32

19. Lezoche E, Baldarelli M, Lezoche G, Paganini AM, Gesuita R, Guerrieri M. Randomized clinical trial of endoluminal locoregional resection versus laparoscopic total mesorectal excision for T2 rectal cancer after neoadjuvant therapy. *BJS*. 2012;99(9):1211-1218. doi:10.1002/bjs.8821

20. Garcia-Aguilar J, Chow OS, Smith DD, Marcet JE, Cataldo PA, Varma MG, Kumar AS, Oommen S, Coutsoftides T, Hunt SR, Stamos MJ, Ternent CA, Herzig DO, Fichera A, Polite BN, Dietz DW, Patil S, Avila K; Timing of Rectal Cancer Response to Chemoradiation Consortium. Effect of adding mFOLFOX6 after neoadjuvant chemoradiation in locally advanced rectal cancer: a multicentre, phase 2 trial. Lancet Oncol. 2015 Aug;16(8):957-66. doi: 10.1016/S1470-2045(15)00004-2. Epub 2015 Jul 14. PMID: 26187751; PMCID: PMC4670237.

21. Rullier E, Rouanet P, Tuech J-J, et al. Organ preservation for rectal cancer (GRECCAR 2): a prospective, randomised, open-label, multicentre, phase 3 trial. *The Lancet*. 2017;390(10093):469-479. doi:10.1016/S0140-6736(17)31056-5

22. Verseveld M, Graaf EJR de, Verhoef C, et al. Chemoradiation therapy for rectal cancer in the distal rectum followed by organ-sparing transanal endoscopic microsurgery (CARTS study). *BJS*. 2015;102(7):853-860. doi:10.1002/bjs.9809

23. Bujko K, Richter P, Smith FM, et al. Preoperative radiotherapy and local excision of rectal cancer with immediate radical re-operation for poor responders: a prospective multicentre study. *Radiother Oncol J Eur Soc Ther Radiol Oncol*. 2013;106(2):198-205. doi:10.1016/j.radonc.2012.12.005

24. van Gijn W, Marijnen CA, Nagtegaal ID, et al. Preoperative radiotherapy combined with total mesorectal excision for resectable rectal cancer: 12-year follow-up of the multicentre, randomised controlled TME trial. *Lancet Oncol*. 2011;12(6):575-582. doi:10.1016/S1470-2045(11)70097-3

25. Marijnen CAM, Velde CJH van de, Putter H, et al. Impact of Short-Term Preoperative Radiotherapy on Health-Related Quality of Life and Sexual Functioning in Primary Rectal Cancer: Report of a Multicenter Randomized Trial. *J Clin Oncol*. September 2016. doi:10.1200/JCO.2005.05.256

26. Moghadamyeghaneh Z, Phelan M, Smith BR, Stamos MJ. Outcomes of Open, Laparoscopic, and Robotic Abdominoperineal Resections in Patients With Rectal Cancer: *Dis Colon Rectum*. 2015;58(12):1123-1129. doi:10.1097/DCR.0000000000000475

27. Smith JJ, Chow OS, Gollub MJ, et al. Organ Preservation in Rectal Adenocarcinoma: a phase II randomized controlled trial evaluating 3-year disease-free survival in patients with locally advanced rectal cancer treated with chemoradiation plus induction or consolidation chemotherapy, and total mesorectal excision or nonoperative management. *BMC Cancer*. 2015;15. doi:10.1186/s12885-015-1632-z

28. Sauer R, Liersch T, Merkel S, et al. Preoperative versus postoperative chemoradiotherapy for locally advanced rectal cancer: results of the German CAO/ARO/AIO-94 randomized phase III trial after a median follow-up of 11 years. *J Clin Oncol Off J Am Soc Clin Oncol*. 2012;30(16):1926-1933. doi:10.1200/JCO.2011.40.1836

29. Fokas E, Liersch T, Fietkau R, et al. Tumor regression grading after preoperative chemoradiotherapy for locally advanced rectal carcinoma revisited: updated results of the CAO/ARO/AIO-94 trial. *J Clin Oncol Off J Am Soc Clin Oncol*. 2014;32(15):1554-1562. doi:10.1200/JCO.2013.54.3769

30. Chua YJ, Barbachano Y, Cunningham D, et al. Neoadjuvant capecitabine and oxaliplatin before chemoradiotherapy and total mesorectal excision in MRI-defined poor-risk rectal cancer: a phase 2 trial. *Lancet Oncol*. 2010;11(3):241-248. doi:10.1016/S1470-2045(09)70381-X

31. Fernandez-Martos C, Brown G, Estevan R, et al. Preoperative Chemotherapy in Patients With Intermediate-Risk Rectal Adenocarcinoma Selected by High-Resolution Magnetic Resonance Imaging: The GEMCAD 0801 Phase II Multicenter Trial. *The Oncologist*. 2014;19(10):1042-1043. doi:10.1634/theoncologist.2014-0233

32. Fernández-Martos C, Pericay C, Aparicio J, et al. Phase II, Randomized Study of Concomitant Chemoradiotherapy Followed by Surgery and Adjuvant Capecitabine Plus Oxaliplatin (CAPOX) Compared With Induction CAPOX Followed by Concomitant Chemoradiotherapy and Surgery in Magnetic Resonance Imaging–Defined, Locally Advanced Rectal Cancer: Grupo Cáncer de Recto 3 Study. *J Clin Oncol*. January 2010. doi:10.1200/JCO.2009.25.8541

33. Dewdney A, Cunningham D, Tabernero J, et al. Multicenter Randomized Phase II Clinical Trial Comparing Neoadjuvant Oxaliplatin, Capecitabine, and Preoperative Radiotherapy With or Without Cetuximab Followed by Total Mesorectal Excision in Patients With High-Risk Rectal Cancer (EXPERT-C). *J Clin Oncol*. 2012;30(14):1620-1627. doi:10.1200/JCO.2011.39.6036

34. Petrelli F, Trevisan F, Cabiddu M, et al. Total Neoadjuvant Therapy in Rectal Cancer: A Systematic Review and Meta-analysis of Treatment Outcomes. *Ann Surg*. 2019;Publish Ahead of Print. doi:10.1097/SLA.0000000000003471

35. Falcone A, Ricci S, Brunetti I, et al. Phase III Trial of Infusional Fluorouracil, Leucovorin, Oxaliplatin, and Irinotecan (FOLFOXIRI) Compared With Infusional Fluorouracil, Leucovorin, and Irinotecan (FOLFIRI) As First-Line Treatment for Metastatic Colorectal Cancer: The Gruppo Oncologico Nord Ovest. *J Clin Oncol*. September 2016. doi:10.1200/JCO.2006.09.0928

36. Masi G, Vivaldi C, Fornaro L, et al. Total neoadjuvant approach with FOLFOXIRI plus bevacizumab followed by chemoradiotherapy plus bevacizumab in locally advanced rectal cancer: the TRUST trial. *Eur J Cancer*. 2019;110:32-41. doi:10.1016/j.ejca.2019.01.006

37. Folkesson J, Birgisson H, Pahlman L, Cedermark B, Glimelius B, Gunnarsson U. Swedish Rectal Cancer Trial: Long Lasting Benefits From Radiotherapy on Survival and Local Recurrence Rate. *J Clin Oncol*. 2005;23(24):5644-5650. doi:10.1200/JCO.2005.08.144

38. Kapiteijn E, Steup WH, Pahlman L, Leer JWH. Preoperative Radiotherapy Combined with Total Mesorectal Excision for Resectable Rectal Cancer. *N Engl J Med*. 2001:9.

39. Bujko K, Wyrwicz L, Rutkowski A, et al. Long-course oxaliplatin-based preoperative chemoradiation versus 5 × 5 Gy and consolidation chemotherapy for cT4 or fixed cT3 rectal cancer: results of a randomized phase III study. *Ann Oncol Off J Eur Soc Med Oncol*. 2016;27(5):834-842. doi:10.1093/annonc/mdw062

40. Bujko K, Nowacki MP, Nasierowska-Guttmejer A, et al. Sphincter preservation following preoperative radiotherapy for rectal cancer: report of a randomised trial comparing short-term radiotherapy vs. conventionally fractionated radiochemotherapy. *Radiother Oncol*. 2004;72(1):15-24. doi:10.1016/j.radonc.2003.12.006

41. Ngan SY, Burmeister B, Fisher RJ, et al. Randomized Trial of Short-Course Radiotherapy Versus Long-Course Chemoradiation Comparing Rates of Local Recurrence in Patients With T3 Rectal Cancer: Trans-Tasman Radiation Oncology Group Trial 01.04. *J Clin Oncol*. 2012;30(31):3827-3833. doi:10.1200/JCO.2012.42.9597

42. Erlandsson J, Holm T, Pettersson D, et al. Optimal fractionation of preoperative radiotherapy and timing to surgery for rectal cancer (Stockholm III): a multicentre, randomised, non-blinded, phase 3, non-inferiority trial. *Lancet Oncol*. 2017;18(3):336-346. doi:10.1016/S1470-2045(17)30086-4

43. Ciseł B, Pietrzak L, Michalski W, et al. Long-course preoperative chemoradiation versus 5 × 5 Gy and consolidation chemotherapy for clinical T4 and fixed clinical T3 rectal cancer: long-term results of the randomized Polish II study. *Ann Oncol*. 2019;30(8):1298-1303. doi:10.1093/annonc/mdz186

44. Myerson RJ, Tan B, Hunt S, et al. Five Fractions of Radiation Therapy Followed by 4 Cycles of FOLFOX Chemotherapy as Preoperative Treatment for Rectal Cancer. *Int J Radiat Oncol*. 2014;88(4):829-836. doi:10.1016/j.ijrobp.2013.12.028

45. Markovina S, Youssef F, Roy A, et al. Improved Metastasis- and Disease-Free Survival With Preoperative Sequential Short-Course Radiation Therapy and FOLFOX Chemotherapy for Rectal Cancer Compared With Neoadjuvant Long-Course Chemoradiotherapy: Results of a Matched Pair Analysis. *Int J Radiat Oncol*. 2017;99(2):417-426. doi:10.1016/j.ijrobp.2017.05.048

46. Nilsson PJ, van Etten B, Hospers GA, et al. Short-course radiotherapy followed by neo-adjuvant chemotherapy in locally advanced rectal cancer – the RAPIDO trial. *BMC Cancer*. 2013;13(1):279. doi:10.1186/1471-2407-13-279

47. Marijnen C. OC-0429: Neoadjuvant chemoradiotherapy or 5x5 Gy followed by chemotherapy in rectal cancer: the RAPIDO trial. *Radiother Oncol*. 2017;123:S227-S228. doi:10.1016/S0167-8140(17)30871-X

48. Francois Y, Nemoz CJ, Baulieux J, et al. Influence of the Interval Between Preoperative Radiation Therapy and Surgery on Downstaging and on the Rate of Sphincter-Sparing Surgery for Rectal Cancer: The Lyon R90-01 Randomized Trial. *J Clin Oncol*. September 2016. doi:10.1200/JCO.1999.17.8.2396

49. Lefevre JH, Mineur L, Kotti S, et al. Effect of Interval (7 or 11 weeks) Between Neoadjuvant Radiochemotherapy and Surgery on Complete Pathologic Response in Rectal Cancer: A Multicenter, Randomized, Controlled Trial (GRECCAR-6). *J Clin Oncol*. 2016;34(31):3773-3780. doi:10.1200/JCO.2016.67.6049

50. Fokas E, Allgäuer M, Polat B, et al. Randomized Phase II Trial of Chemoradiotherapy Plus Induction or Consolidation Chemotherapy as Total Neoadjuvant Therapy for Locally Advanced Rectal Cancer: CAO/ARO/AIO-12. *J Clin Oncol*. May 2019:JCO.19.00308. doi:10.1200/JCO.19.00308

51. Maas M, Lambregts DMJ, Nelemans PJ, et al. Assessment of Clinical Complete Response After Chemoradiation for Rectal Cancer with Digital Rectal Examination, Endoscopy, and MRI: Selection for Organ-Saving Treatment. *Ann Surg Oncol*. 2015;22(12):3873-3880. doi:10.1245/s10434-015-4687-9

52. Battersby NJ, Dattani M, Rao S, et al. A rectal cancer feasibility study with an embedded phase III trial design assessing magnetic resonance tumour regression grade (mrTRG) as a novel biomarker to stratify management by good and poor response to chemoradiotherapy (TRIGGER): study protocol for a randomised controlled trial. *Trials*. 2017;18. doi:10.1186/s13063-017-2085-2

53. Patel UB, Blomqvist LK, Taylor F, et al. MRI After Treatment of Locally Advanced Rectal Cancer: How to Report Tumor Response—The MERCURY Experience. *Am J Roentgenol*. 2012;199(4):W486-W495. doi:10.2214/AJR.11.8210

54. Patel UB, Taylor F, Blomqvist L, et al. Magnetic Resonance Imaging–Detected Tumor Response for Locally Advanced Rectal Cancer Predicts Survival Outcomes: MERCURY Experience. *J Clin Oncol*. August 2011. doi:10.1200/JCO.2011.34.9068

55. Patel UB, Brown G, Rutten H, et al. Comparison of Magnetic Resonance Imaging and Histopathological Response to Chemoradiotherapy in Locally Advanced Rectal Cancer. *Ann Surg Oncol*. 2012;19(9):2842-2852. doi:10.1245/s10434-012-2309-3

56. Siddiqui MRS, Gormly KL, Bhoday J, et al. Interobserver agreement of radiologists assessing the response of rectal cancers to preoperative chemoradiation using the MRI tumour regression grading (mrTRG). *Clin Radiol*. 2016;71(9):854-862. doi:10.1016/j.crad.2016.05.005

57. Horvat N, Carlos Tavares Rocha C, Clemente Oliveira B, Petkovska I, Gollub MJ. MRI of Rectal Cancer: Tumor Staging, Imaging Techniques, and Management. *RadioGraphics*. 2019;39(2):367-387. doi:10.1148/rg.2019180114

58. Nahas SC, Rizkallah Nahas CS, Sparapan Marques CF, et al. Pathologic Complete Response in Rectal Cancer: Can We Detect It? Lessons Learned From a Proposed Randomized Trial of Watch-and-Wait Treatment of Rectal Cancer. *Dis Colon Rectum*. 2016;59(4):255-263. doi:10.1097/DCR.0000000000000558

59. van der Sande ME, Beets GL, Hupkens BJP, et al. Response assessment after (chemo)radiotherapy for rectal cancer: Why are we missing complete responses with MRI and endoscopy? *Eur J Surg Oncol*. 2019;45(6):1011-1017. doi:10.1016/j.ejso.2018.11.019

60. Bettegowda C, Sausen M, Leary RJ, et al. Detection of Circulating Tumor DNA in Early- and Late-Stage Human Malignancies. :13.

61. Tie J, Wang Y, Tomasetti C, et al. Circulating tumor DNA analysis detects minimal residual disease and predicts recurrence in patients with stage II colon cancer. *Sci Transl Med*. 2016;8(346):346ra92-346ra92. doi:10.1126/scitranslmed.aaf6219

62. Thorsteinsson M, Jess P. The clinical significance of circulating tumor cells in non-metastatic colorectal cancer – A review. *Eur J Surg Oncol EJSO*. 2011;37(6):459-465. doi:10.1016/j.ejso.2011.01.025

63. Suwinski R, Wzietek I, Tarnawski R, et al. Moderately Low Alpha/Beta Ratio for Rectal Cancer May Best Explain the Outcome of Three Fractionation Schedules of Preoperative Radiotherapy. *Int J Radiat Oncol*. 2007;69(3):793-799. doi:10.1016/j.ijrobp.2007.03.046

64. Venook AP. NCCN Guidelines Index Table of Contents Discussion. *Rectal Cancer*. 2019:167.

65. Temple LKMD, Bacik JMS, Savatta SGMD, et al. The Development of a Validated Instrument to Evaluate Bowel Function After Sphincter-Preserving Surgery for Rectal Cancer. *Dis Colon Rectum*. 2005;48(7):1353-1365. doi:10.1007/s10350-004-0942-z

66. Emmertsen KJ, Laurberg S. Impact of bowel dysfunction on quality of life after sphincter-preserving resection for rectal cancer. *BJS*. 2013;100(10):1377-1387. doi:10.1002/bjs.9223

67. Emmertsen KJ, Laurberg S. Low Anterior Resection Syndrome Score: Development and Validation of a Symptom-Based Scoring System for Bowel Dysfunction After Low Anterior Resection for Rectal Cancer. *Ann Surg*. 2012;255(5):922-928. doi:10.1097/SLA.0b013e31824f1c21

68. Baxter NNMD, Novotny PJMS, Jacobson TRN, Maidl LJRN, Sloan J, Young-Fadok TMMD. A Stoma Quality of Life Scale. *Dis Colon Rectum*. 2006;49(2):205-212. doi:10.1007/s10350-005-0275-6

69. Rosen RC, Riley A, Wagner G, Osterloh IH, Kirkpatrick J, Mishra A. The international index of erectile function (IIEF): a multidimensional scale for assessment of erectile dysfunction. *Urology*. 1997;49(6):822-830. doi:10.1016/S0090-4295(97)00238-0

70. Rosen RC, Cappelleri JC, Smith MD, Lipsky J, Peña BM. Development and evaluation of an abridged, 5-item version of the International Index of Erectile Function (IIEF-5) as a diagnostic tool for erectile dysfunction. *Int J Impot Res*. 1999;11(6):319-326. doi:10.1038/sj.ijir.3900472

71. R. Rosen RD C Brown, J Heiman, S Leiblum, C Meston, R Shabsigh, D Ferguson. The Female Sexual Function Index (FSFI): A Multidimensional Self-Report Instrument for the Assessment of Female Sexual Function. *J Sex Marital Ther*. 2000;26(2):191-208. doi:10.1080/009262300278597

72. USRF - International Prostate Symptom Score. http://www.usrf.org/questionnaires/AUA_SymptomScore.html. Accessed November 21, 2019.

73. Herdman M, Gudex C, Lloyd A, et al. Development and preliminary testing of the new five-level version of EQ-5D (EQ-5D-5L). *Qual Life Res*. 2011;20(10):1727-1736.

74. Dattani M, Heald R, Goussous G, et al. Oncological and Survival Outcomes in Watch and Wait Patients With a Clinical Complete Response After Neoadjuvant Chemoradiotherapy for Rectal Cancer: A Systematic Review and Pooled Analysis. *Ann Surg*. 2018;268(6):955-967. doi:10.1097/SLA.000000000000276
